# Supplementary material for: Genetic or pharmacological GHSR blockade has sexually dimorphic effects in rodents on a high-fat diet
Source: Commun Biol. 2024 May 25;7:632. doi: 10.1038/s42003-024-06303-5 (PMC11127961; doi:10.1038/s42003-024-06303-5)
Supplement: Supplementary file 2 — Supplementary Information [file 42003_2024_6303_MOESM2_ESM.pdf]

Genetic or pharmacological GHSR blockade has sexually dimorphic effects in rodents on a high-fat diet

Andras H. Leko[1,2*], Adriana Gregory-Flores[1,3*], Renata C. N. Marchette[3*], Juan L. Gomez[4], Janaina C.M. Vendruscolo[3], Vez Repunte-Canonigo[5], Vicky Chuong[1,3], Sara L. Deschaine[1], Kimberly Whiting[1,3], Shelley N. Jackson[6], Maria Paula Cornejo[7], Mario Perello[7], Zhi-Bing You[8], Michael Eckhaus[9], Karuna Rasineni[10,11,12], Kim D. Janda[13], Barry Zorman[14], Pavel Sumazin[14], George F. Koob[3], Michael Michaelides[4], Pietro P. Sanna[5], Leandro F. Vendruscolo[15#], Lorenzo Leggio[1,6,16,17,18#]

SUPPLEMENTARY INFORMATION

MATERIAL AND METHODS

BEHAVIORAL ASSESSMENTS

Given that locomotion and anxiety-like behavior may affect feeding and the orexigenic actions of ghrelin, we tested GHSR-KO and WT rats in an open field test and a novelty-suppressed feeding test.

Open Field Test

The open field test under low light conditions (and small space) is commonly used to assess spontaneous activity and locomotion in rodents[1]. We investigated the effects of HFD and GHSR

deletion on locomotor activity after 40 weeks of *ad libitum* access to either chow or high-fat diet (HFD). Rats were habituated to the testing room for at least 1 h prior to the open field test. The order of testing was randomly selected based on genotype and diet assignments. Males and females were tested on separate days. Rats were placed in the center of a dark, gray small open field (40 x 40 x 50 cm) and ambulation was recorded for 5 min. The apparatus was cleaned with Labsan 256 CPQ (Sanitation Strategies LLC, Holt, MI) after each rat completed the task. The videos were analyzed for total distance moved using EthoVision tracking software (Noldus, Leesburg, VA, USA).

Novelty-Suppressed Feeding Test

This test is commonly used to assess anxiety-like behavior[2]. After 18 weeks of *ad libitum* access to either chow or high-fat diet (HFD), rats were food deprived for 23 h prior to the test; water was available *ad libitum* during this time. The rats were habituated to an anteroom for at least 1 h and carried into the testing room at the beginning of the test. The order of testing was randomly selected based on genotype and diet assignments. Males and females were tested on separate days. Chow or HFD was placed in the center of a large (100 x 100 x 30 cm), white open field depending on the rats' diet assignment (i.e., chow or HFD). Rats were placed in one of the corners facing the center of the open field. Latency to feed was recorded from the moment that the rat was placed in the open field until it began to bite into the food (i.e., not just sniffing or holding the food). At this point, the rat was removed from the apparatus and returned to its home cage. The maximum testing time was 10 min, when the rat was removed from the open field if it did not reach the food. The apparatus was cleaned with Labsan 256 CPQ (Sanitation Strategies LLC, Holt,

MI) after each rat completed the test. After the test, food consumption in the home cage was recorded for 60 min to determine any differences in appetite between the groups in a low-stress environment (i.e., home cages), after which *ad libitum* access to chow or HFD was reinstated.

LIVER HISTOLOGY

Liver tissue samples were sent to Histoserv Inc. (Germantown, Maryland) where tissue was paraffin processed and stained with hematoxylin-eosin[5]. The slides were then evaluated by a veterinary pathologist, using a qualitative steatosis scoring to evaluate the lipid content of hepatocytes by microscopic analysis. Two livers were used from each group with the same genotype-sex-diet. Steatosis was scored on an 8-point scale: 0 – negative, 1 – minimal, 2 – minimal to mild, 3 – mild, 4 – mild to moderate, 5 – moderate, 6 – moderate to severe, 7 – severe.

MEASUREMENT OF HEPATIC TRIGLYCERIDES

Hepatic lipids were extracted following the Folch procedure {Folch, 1957 #14}. Aliquots of lipid extracts were subjected to saponification and then the triglyceride levels were measured using the triglyceride diagnostics kit (Thermo Fisher Scientific, Middletown, VA).

ENDOCRINE ASSAYS

To characterize the endocrine and metabolic profile of GHSR-KO and WT rats, we performed glucose tolerance and insulin tolerance tests. Blood levels of several hormones were also measured at the end of the experiments (terminal hormone analysis, described further below).

For endocrine assessments, tail blood collections were performed. Briefly, the experimenter gently held the rat to snip the tip of the tail and collect blood (~200 µl). The procedure

was quick (less than 1 min) and anesthesia was not necessary. Hemostasis was achieved by gently drying the tail tip with paper towel. For multiple blood collections in a single day, the tail was only snipped once for the first collection. Subsequent collections were achieved by gently squeezing the tail from the base to the tip until blood flow was reestablished.

Glucose Tolerance Test

On week 51 after initiation of Chow or HFD, following a 12 h overnight food-deprivation, rats received an intraperitoneal (IP) injection of a 20% (w/v) glucose solution in saline (1 g/kg) 4-6 h into the light cycle. Glucose levels were measured at baseline and at 20-, 60- and 120-min post-glucose injection. Blood glucose was measured with a commercially available Aimstrip Plus blood glucose monitor (Germaine Laboratories, San Antonio, TX) at the time of the blood collection. Plasma insulin levels were measured at baseline and at 60 min post-glucose injection. Plasma insulin concentrations were analyzed using a commercial rat/mouse Insulin ELISA kit (EMD Millipore, Billerica, MA) and assayed in duplicates according to manufacturer instructions.

Insulin Tolerance Test

On week 52 after initiation of Chow or HFD, following a 12 h overnight food-deprivation, rats received an IP injection of insulin (1 U/kg) 4-6 h into the light cycle. Glucose levels were measured at baseline and at 20-, 60- and 120-min post-insulin injection. Blood glucose levels were measured as described above.

Terminal Hormone Analysis

At the time of the carcass analysis (see Methods in the main manuscript for additional details), blood was collected through cardiac puncture in EDTA coated Eppendorf tubes with or without the ghrelin inhibitor Pefabloc ® SC (Cat #11429876001, Millipore Sigma, Billerica, MA; 1mg/mL final concentration). Blood samples were immediately centrifuged at 2,000 x g for 15 min at 4°C. Plasma extracted from blood samples with ghrelin inhibitor Pefabloc® SC were treated with 5% (v/v) 1M HCl before storage at -80 °C. Plasma extracted from blood samples without ghrelin inhibitors were stored at -80 °C until assay. The various plasma hormone concentrations were analyzed using commercial rat/mouse ELISA kits for ghrelin (acylated form) and desacyl-ghrelin (ALPCO, Macedon, NY), insulin, leptin, and growth hormone (GH) ELISA kits (EMD Millipore, Billerica, MA), corticosterone and aldosterone ELISA kits (Abcam, Cambridge, MA), progesterone and testosterone ELISA kits (Cayman Chemical, Ann Harbor, MI), and a LEAP2 ELISA kit (MyBioSource, San Diego, CA). All samples were assayed in duplicates according to manufacturer instructions.

ANTI-GHRELIN VACCINE EXPERIMENT IN MALE WISTAR RATS

Animals

Given the results of the GHSR KO in diet-induced obesity (DIO), indicating an effect of HFD in male but not female rats (present study; see **Results**), we limited this experiment to male rats only. The rats were exposed to HFD only (no Chow condition was included). Sixteen adult male Wistar rats were obtained from Charles River (Kingston, New York, NY, USA). The rats weighed 250-275 g at the beginning of the study. All animals were single-housed and maintained under a 12 h/12 h light/dark cycle (lights off at 6:00PM) at 21ºC ± 2ºC with *ad libitum* access to food and water. All procedures were conducted per the National Institutes of Health Guide for the

Care and Use of Laboratory Animals and were approved by the National Institute on Drug Abuse, Intramural Research Program, Animal Care and Use Committee.

Vaccine Preparation

The active Ghr1-TT-Alum-CpG ODN vaccine formulation for injection was composed of a 5:1:5 (v/v/v) mixture of A) Gherlin-Thr-Thr (Ghr1-TT) immunoconjugate (1.25 mg/ml in PBS), B) CpG oligodeoxynucleotides (ODN) 1826 (1.25 mg/ml in PBS), and C) Imject Alum (5 mg/ml in PBS). For each active injection, one aliquot each of Ghr1-TT, CpG ODN 1826, and Alum were diluted to the correct concentrations, then mixed together to form the vaccine formulation, and shaken at room temperature for 30 min. After shaking, the vaccine was stored on ice for no more than 1 h prior to injection. The control TT-Alum-CpG ODN 1826 vaccine formulation for injection was generated through the same procedure, with a 5:1:5 (v/v/v) mixture of TT (1.25 mg/mL in PBS), CpG ODN 1826 (1.25 mg/mL in PBS), and Imject Alum (5 mg/mL in PBS).

Vaccination Procedure

The rats were individually housed in the vivarium facility for 1 week prior to the initiation of study. Each rat was injected IP with 400 µL of vaccine formulation, delivering a dose of 250 µg Ghr1-TT, 50 µg CpG ODN 1826, and 1 mg of Alum per animal for each injection. The rats were injected with a priming dose on week 0, with boosts administered on weeks 3, 5, 8, 12 and 17. Tail bleeds were taken on weeks 4, 6, 9, 13 and 18. We recorded body weight and food intake throughout the experiment.

ELISA Test analysis for the assessment of the midpoint titer

Production of anti-ghrelin Ig was evaluated by ELISA. Microtiter plates (Costar 3690) were incubated with coating antigen Ghr1-BSA in PBS (5 µg/mL, 25 µL) (18 h, 37ºC). 5% Non-fat milk in PBS (30 min, 25ºC) was added to block non-specific binding. Rat sera were diluted

1:100 in 2% BSA in PBS then serially diluted across the plates (1:2) before incubation in a moist chamber (1 h, 37ºC). Plates were washed 10 times with H_2O before incubation with peroxidase-conjugated donkey anti-rat IgG (Jackson ImmunoResearch), mouse anti-rat IgM (bleeds 1 – 3), or goat anti-rat Ig (Southern Biotech, Inc.) in a moist chamber (30 min, 37ºC). The plates were further washed 10 times with H_2O before being developed with the TMB (3,3',5,5' tetramethylbenzidine dihydrochloride) substrate kit (Thermo Pierce) and the absorbance at 450 nm measured on a microplate reader (SpectraMax M2 Molecular Devices). Titers were calculated as the dilution corresponding to 50% of the maximum absorbance from a plot of the absorbance versus log(dilution) using GraphPad Prism 6.

Analysis of Antibody Binding Affinity by Surface Plasmon Resonance

Production of anti-Ghrelin Ig was evaluated by Surface Plasmon Resonance (SPR) methodology. A Biacore 3000 instrument equipped with a CM5 sensor chip (GE Healthcare) was used for all the analyses. Reference flow cell 1 was immobilized with ~5,000 RU of BSA, and flow cell 2 was immobilized with ~5,000 RU of Ghr1-BSA using NHS/EDC chemistry according to the manufacturer's instruction. All assays were conducted at 25ºC with a flow rate of 30 µL/min, using HBS-EP+ buffer as running buffer. The surface was regenerated with 50 mM NaOH for 30 sec at 30 µL/min. All sera were titrated, and the concentration was normalized to give a response of ~100 RU in the assay. To determine the IC50 of rat full length ghrelin (rflGhre) for vaccinated sera, individual serum was incubated at RT for 1hr with various concentration of rflGhre, ranging from 1 µM-0.98 nM, then were passed over the flow cells. For control-generated sera, only the two highest concentration of rflGhre (1 and 0.5 µM) were used in competition assay. An anti-ghrelin control monoclonal antibody (JG4) was also used in IC50 determination as a positive control. IC50 were calculated using GraphPad Prism 6.

Diets

Rats were fed a HFD that was introduced 1 week following treatment with the priming dose on week 0 and consisted of 20% protein, 20% carbohydrate and 60% fat by kcal (Research Diets D12492, New Brunswick, NJ).

Novelty-Suppressed Feeding Test

This test is used to assess anxiety-like behavior[2] and was done on week 15 after the completion of the vaccination procedure. Rats were food deprived for 23 h with *ad libitum* access to water. Then, the rats were placed in an open field (100 x 100 x 30 cm) facing one of the corners and left to explore for 10 min. HFD pellets were placed in the center of the open field. Latency to feed was recorded from the moment the rat was placed in the open field until it began to consume the HFD, not just merely sniffing or holding the food. At this point, the rat was removed from the apparatus and returned to its home cage. Food consumption in the home cage was recorded over the next 60 min, after which *ad libitum* access HFD was reinstated.

Blood Glucose Concentrations

A razor blade was used to snip the tip of the tail (1 mm) to collect blood. The experimenter gently held the rat to snip the tip of the tail and collect blood. The procedure was quick (less than 2 min) and anesthesia was not necessary. Hemostasis was achieved by gently drying the tail tip with a paper towel. Blood glucose samples were collected on week 16 after completion of the vaccination procedure. Blood was collected within the 1st h after the start of the light cycle (lights on at 6:00AM) on separate days during fasting and satiated conditions._Blood glucose was measured with a commercially available Aimstrip Plus blood glucose monitor (Germaine Laboratories, San Antonio, TX).

Statistical Analysis

All data were expressed as means and standard errors of the mean (SEM). Data were analyzed using analysis of variance (ANOVA) with repeated measures and multiple unpaired *t*-test analysis. Mauchly's test was used to assess the assumption of sphericity. If that was violated for the main effect of time, the degree of freedom was corrected using Greenhouse-Geisser estimate of sphericity. When appropriate, *post hoc* comparisons were performed using Bonferroni or Duncan's tests for multiple comparisons. Statistical significance for all analyses was set at $p \leq 0.05$. GraphPad Prism 9.0.1 was used for statistical analysis.

ANTI-GHRELIN VACCINE EXPERIMENT IN MALE AND FEMALE C57BL/6J MICE

Animals

Given the previous results in non-obese male mice[12] and the anti-ghrelin vaccine experiment in male rats exposed to HFD described here (see Supplemental Results above in this Supplement), we wondered whether species and/or sex differences could account for these differences. Therefore, we performed another anti-ghrelin vaccine experiment in male and female mice. Male (n = 16) and female (n = 16) C57BL/6J mice were obtained from Jackson's Laboratory (Bar Harbor, ME, USA), and were 6 weeks-old upon arrival. All mice were single housed in standard cages and had *ad libitum* access to food and water. Mice were maintained in a temperature and humidity-controlled room with a 12h /12 h dark/light cycle (lights off 7:00AM).

Vaccine Preparation

Mice were treated with a Ghr1-CRM-Alum-CpG ODN vaccine. The Ghr1 peptide was conjugated to the carrier protein Diphtheria Toxoid mutant CRM 197, a non-toxic mutant used to make polysaccharides and haptens immunogenic, and to a Bovine Serum Albumin carrier for ELISA analysis. This step is necessary to ensure the immunogenicity of the Ghr1 hapten. The

active vaccine formulation was prepared with a 5:1:5 (v/v/v) mixture of A) Ghr1-CRM immunoconjugate (1.25 mg/ml in PBS), B) CpG ODN 1826 (1.25 mg/ml in PBS), and C) Alhydrogel® (5 mg/ml in PBS). Once all components were combined, the active vaccine was shaken for 30 min at room temperature. The vaccine was kept on ice for no more than 1 h prior to injection. The sham vaccine was prepared the same as active vaccine, but instead formulated with a 5:1:5 (v/v/v) mixture of A) A genetically modified cross-reacting material (CRM) of diphtheria toxin carrier protein (1.25 mg/ml in PBS), B) CpG ODN 1826 (1.25 mg/ml in PBS), and C) Alhydrogel® (5 mg/ml in PBS).

Vaccination Procedure

Mice were habituated for 1 week upon arrival in the vivarium facility and prior to initiation of the study. Mice were treated with either active or sham vaccine intraperitoneally at a volume of 110 μL. Each dose delivered 50 μg of Ghr1-CRM or unconjugated CRM, 50 μg CpG ODN 1826, and 150 μg of Alhydrogel® per mouse. A priming dose was administered on week 0, followed by vaccine boosts on weeks 3, 5 and 8. Blood was collected from mice via the submandibular facial vein for determination of IgG response towards the vaccine. Bleeds were conducted one week following administration of vaccine boosts on weeks 4, 6 and 9. Blood was not centrifuged down to collect the sera, but was instead frozen at -20ºC as whole blood, which precluded the use of Biacore for affinity analysis. All blood was analyzed by ELISA to determine IgG titers, and IgM titers were examined for a select number of mice.

Diets

Mice were separated into two groups with *ad libitum* access to either regular chow or high-fat diet (HFD). HFD was introduced 1 week following treatment with the priming dose on week 0

and consisted of 20% protein, 20% carbohydrate and 60% fat by kcal (Research Diets D12492, New Brunswick, NJ). Chow and HFD intake and body weight was measured once per week.

Statistical Analysis

We analyzed regular chow and HFD separately. Weekly food consumption and body weight were analyzed by a three-way repeated measures ANOVA. Then, since no significant sex effects were identified, we combined male and female data to increase the power of the analysis and performed a two-way repeated measures ANOVA with treatment and time as independent factors. Mauchly's test was used to assess the assumption of sphericity. If that was violated for the main effect of time, the degree of freedom was corrected using Greenhouse-Geisser estimate of sphericity. When appropriate ($p < 0.05$), Bonferroni *post hoc* test was used for comparisons.

RESULTS

FEED EFFICIENCY DURING A HIGH FAT DIET

We calculated the weekly feed efficiency (weight gain [mg] / energy consumption [kcal]) during HFD. A two-way RM ANOVA did not detect a significant main effect of Genotype on the feed efficiency, only a significant effect of Time in males ($F_{2.065, 26.85} = 41.10$, $p < 0.0001$; **Supplemental Figure S1A**) and females ($F_{4.475, 62.65} = 12.76$, $p < 0.0001$; **Supplemental Figure S1B**).

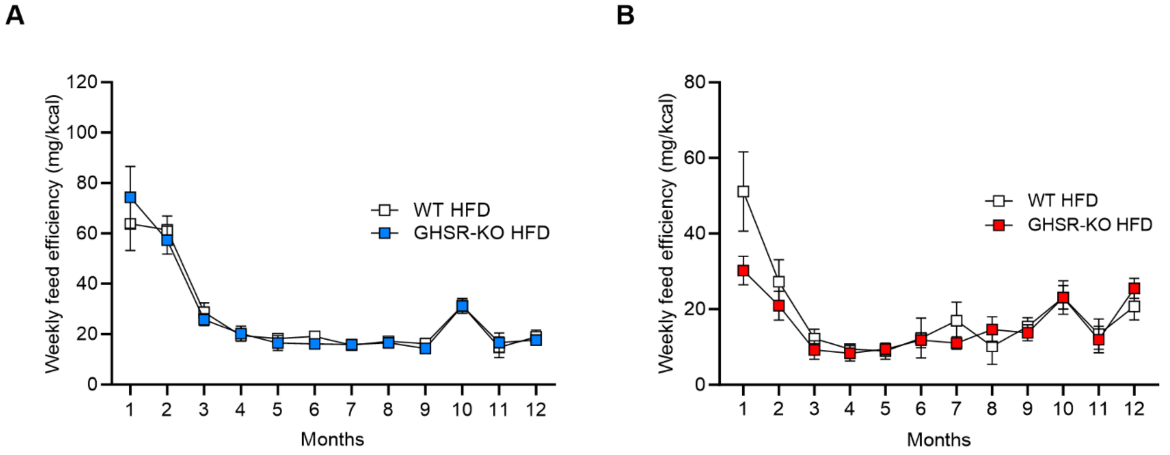

Supplemental Figure S1. No differences in weekly feed efficiency (weight gain [mg] / energy consumption [kcal]) between WT and GHSR-KO male and female rats fed with HFD. (**A**) There was no significant difference between WT and GHSR-KO male rats fed with HFD in weekly feed efficiency. WT: $n = 7$, GHSR-KO: $n = 8$ (**B**) There was no significant difference between WT and GHSR-KO female rats fed with HFD in weekly feed efficiency. WT: $n = 6$, GHSR-KO: $n = 10$.

BEHAVIORAL ASSESSMENTS

Open Field Test

The ANOVA did not reveal significant main effects of Diet or Genotype nor interactions in locomotion in either male (**Supplemental Figure S2A**) or female (**Supplemental Figure S2B**) rats as measured by distance travelled in the open field.

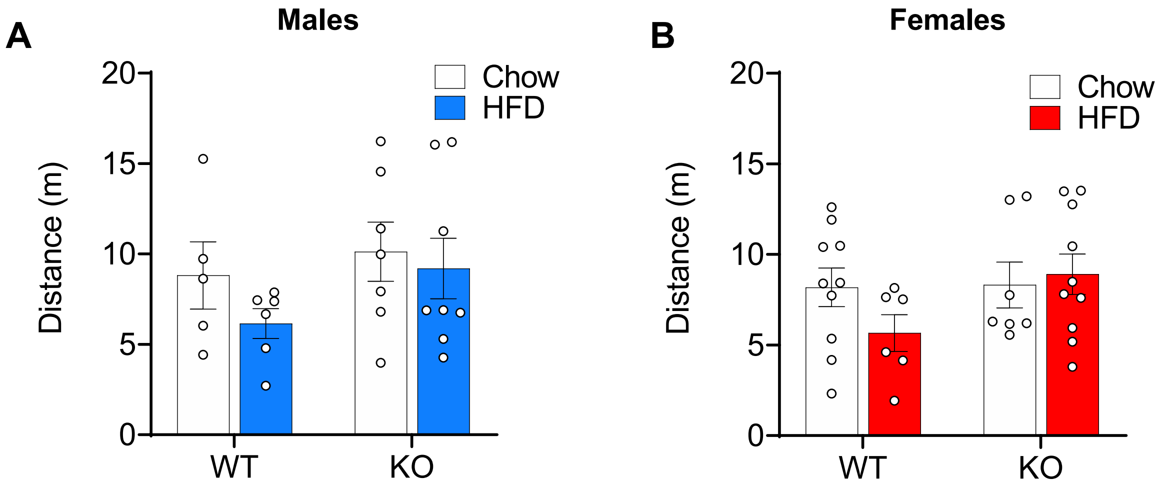

Supplemental Figure S2. No differences in locomotion between WT and GHSR-KO male and female rats fed with either HFD or chow. (**A**) There was no significant difference between WT and GHSR-KO male rats fed with either HFD or chow in distance travelled in the open field. WT: n = 5 Chow, n = 6 HFD. GHSR-KO: n = 7 Chow, n = 8 HFD. (**B**) There was no significant difference between WT and GHSR-KO female rats fed with either HFD or chow in distance travelled in the open field. WT: n = 10 Chow, n = 6 HFD. GHSR-KO: n = 7 Chow, n = 10 HFD.

Novelty-Suppressed Feeding Test

The ANOVA did not reveal main effects of genotype or diet, nor interactions between these two factors for the time to reach food in the novel (stressful) arena in males (**Supplemental Figure S3A**) or females (**Supplemental Figure S3B**). Moreover, there were no statistically significant differences in food consumption during 1 h in male rats upon return to the home-cage (low stress environment; **Supplemental Figure S3C**). However, there was a main effect of Diet ($F_{1, 33}$ = 4.580, $p < 0.05$) on 1 h food consumption in the home-cage in female rats (**Supplemental Figure S3D**), indicating that HFD-fed female rats consumed less calories during 1 h after the novelty suppressed feeding test than chow-fed female rats, regardless of genotype.

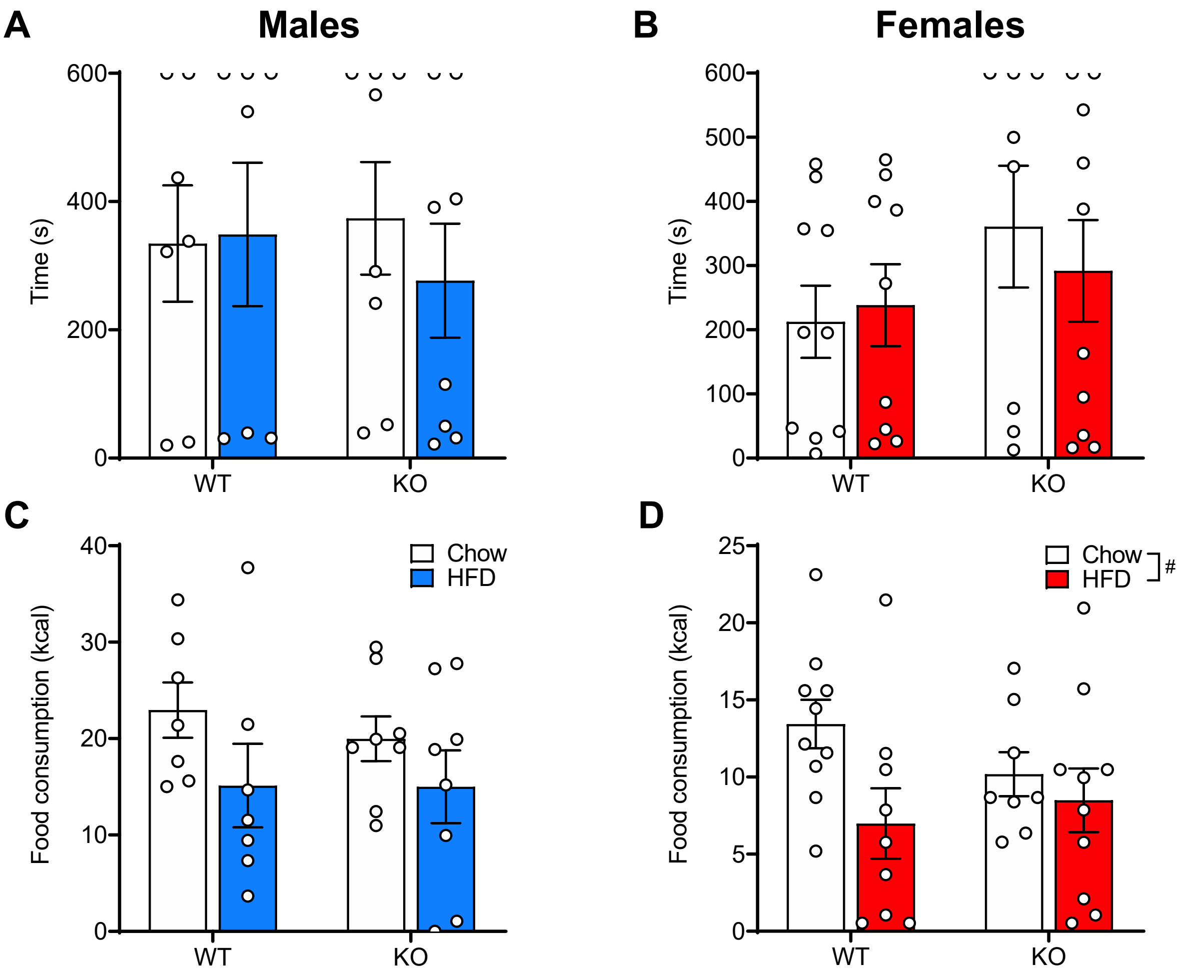

Supplemental Figure S3. GHSR deletion has no effect on novelty-suppressed feeding. (**A**) There was no significant difference in latency to feed in a novel arena between WT and GHSR-KO male rats, regardless of diet. (**B**) There was no significant difference in latency to feed in a novel arena between WT and GHSR-KO female rats, regardless of diet. (**C**) One hour food consumption in the home-cage did not differ between WT and GHSR-KO male rats. WT: $n = 7$ Chow, $n = 7$ HFD. GHSR-KO: $n = 7$ Chow, $n = 8$ HFD. (**D**) Overall, HFD-fed female rats consumed less calories during 1-hour food consumption in the familiar home-cage than chow-fed rats ($^{\#}p < 0.05$), regardless of genotype. WT: $n = 10$ Chow, $n = 6$ HFD. GHSR-KO: $n = 7$ Chow, $n = 10$ HFD.

CARCASS ANALYSIS

The 2-way ANOVA did not indicate any significant effects of Diet or Genotype on the percent of BAT weight, in both sexes (**Supplemental Figure S4A-B**). In males, we found a significant effect of Diet ($F_{1, 24} = 88.06$, $p < 0.0001$; HFD > Chow) and Genotype ($F_{1, 24} = 6.489$, $p < 0.05$; GHSR-KO < WT) for the percent of gonadal WAT weight (**Supplemental Figure S4C**),

and a significant effect of Diet ($F_{1, 29} = 6.907$, $p < 0.05$; HFD > Chow) in females (**Supplemental Figure S4D**). The 2-way ANOVA indicated an effect of Diet ($F_{1, 24} = 19.69$, $p < 0.001$; HFD > Chow) on the percent of inguinal WAT weight in males, but not females (**Supplemental Figure S4E-F**). We did not find significant effects for gonadal/inguinal WAT ratio, in both sexes (**Supplemental Figure S4G-H**).

In males, there were no significant main effects or interactions of Diet and Genotype on nose-anus length, nor on percentage of adrenal glands in body weight **(Supplemental Table 1**). The 2-way ANOVA indicated a significant effect of Diet on spleen weight ($F_{1, 24} = 10.96$, $p < 0.01$; Chow > HFD).

The 2-way ANOVA showed an effect of Diet on the percentage of liver weight ($F_{1, 24} = 179.7$, $p < 0.0001$), indicating decreased liver weights in HFD-fed male rats compared with chow-fed male rats, regardless of genotype (**Supplemental Table 1**).

In female rats, the 2-way ANOVA indicated a similar difference in the percentage of liver weight, namely, the ratio of liver weight in the total body weight was higher in chow-fed animals, regardless of genotype ($F_{1, 29} = 24.11$, $p < 0.0001$). ANOVAs indicated no significant main effects or interactions of Diet and Genotype for nose-anus length, nor for the percentage of spleen weights, and adrenal gland weights (**Supplemental Table 1**).

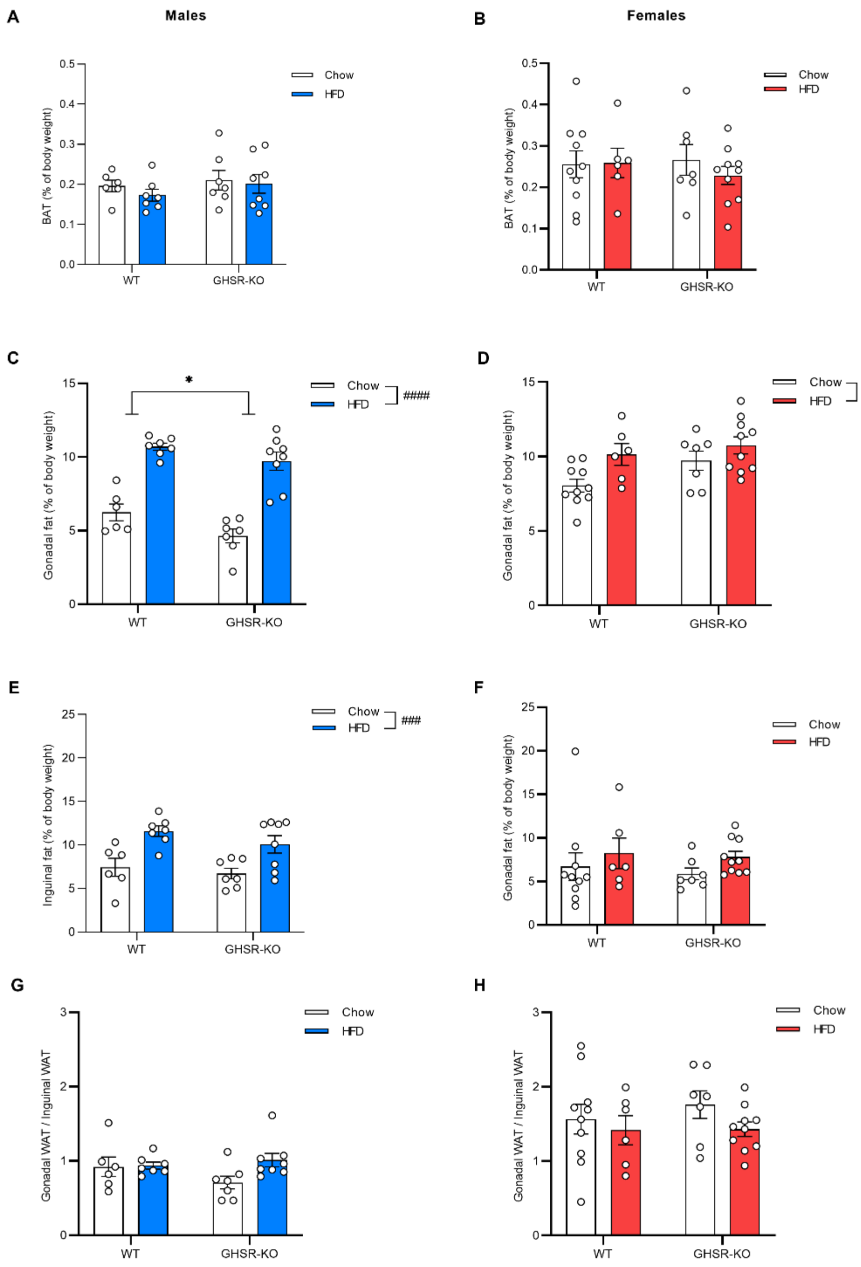

Supplemental Figure S4. GHSR deletion results in less percentage of gonadal WAT in total body weight in male, but not female, rats. (**A**) GHSR knockout (KO) male rats and WT male rats fed with a HFD or a chow diet did not differ in relative BAT weight (% of body weight). WT chow: $n = 6$. GHSR-KO chow: $n = 7$. WT HFD: $n = 7$. GHSR-KO HFD: $n = 8$. (**B**) There were no genotype or diet effects in female rats on relative BAT weight. WT chow: $n = 10$. GHSR-KO chow: $n = 7$. WT HFD: $n = 6$. GHSR-KO HFD: $n = 10$. (**C**) WT male rats had more gonadal fat compared with GHSR-KO male rats ($^{*}p < 0.05$). HFD-fed rats had more gonadal fat compared to chow-fed rats ($^{\#\#\#\#}p < 0.0001$) (**D**) Female rats that received HFD had a higher percentage of gonadal fat than the female rats fed a regular chow diet, regardless of genotype ($^{\#}p < 0.05$). (**E**) There was no genotype effect on the percentage of inguinal fat in male rats. HFD-fed males had more inguinal fat than chow-fed males, regardless of genotype ($^{\#\#\#}$ $p < 0.001$). (**F**) There were no genotype or diet effects in female rats on relative inguinal fat weight. (**G**) We observed no effect of Diet or Genotype on gonadal/inguinal WAT ratio in males (**H**) There were no Genotype or Diet effects in female rats on gonadal/inguinal WAT ratio. Data are expressed as mean ± SEM, circles represent each individual rat.

Supplemental Table 1. Results of the carcass analysis on nose-anus length and on relative weights (% of body weight) of adrenal, spleen, and liver of wild-type (WT) or GHSR knock-out (KO) male and female rats fed with chow or a high-fat diet (HFD).

**Treatment group**	**Nose-anus length (cm)**	**Adrenal glands (% of BW)**	**Spleen (% of BW)**	**Liver (% of BW)**
Male WT Chow (6)	28.4 ± 0.45	0.011 ± 0.0007	0.164 ± 0.009##	2.651 ± 0.079####
Male GHSR-KO Chow (7)	28.3 ± 0.34	0.012 ± 0.002	0.170 ± 0.020##	2.519 ± 0.077####
Female WT Chow (10)	23.2 ± 0.28	0.017 ± 0.001	0.167 ± 0.010	2.413 ± 0.104####
Female GHSR-KO Chow (7)	23.1 ± 0.23	0.017 ± 0.002	0.163 ± 0.023	2.234 ± 0.085####
Male WT HFD (7)	29.2 ± 0.44	0.008 ± 0.001	0.137 ± 0.010	1.706 ± 0.039
Male GHSR-KO HFD (8)	29.0 ± 0.38	0.011 ± 0.001	0.114 ± 0.008	1.815 ± 0.049
Female WT HFD (6)	23.4 ± 0.35	0.014 ± 0.003	0.166 ± 0.005	1.866 ± 0.122
Female GHSR-KO HFD (10)	24.1 ± 0.29	0.011 ± 0.001	0.140 ± 0.011	1.836 ± 0.066

Data are presented as mean ± standard error of the mean (SEM). The number of samples tested are noted in parentheses.
##Difference from HFD-fed rats of the same sex, regardless of genotype ($p < 0.01$)
####Difference from HFD-fed rats of the same sex, regardless of genotype ($p < 0.0001$)

LIVER HISTOLOGY

Liver samples of HFD-fed male and female rats showed mild-to-severe steatosis (scores 4-7), while those of chow-fed animals in both sexes showed either no sign or only mild-to-moderate steatosis (scores 0-5). We did not observe any difference in steatosis related to genotype or sex **(Supplemental Figure S5)**.

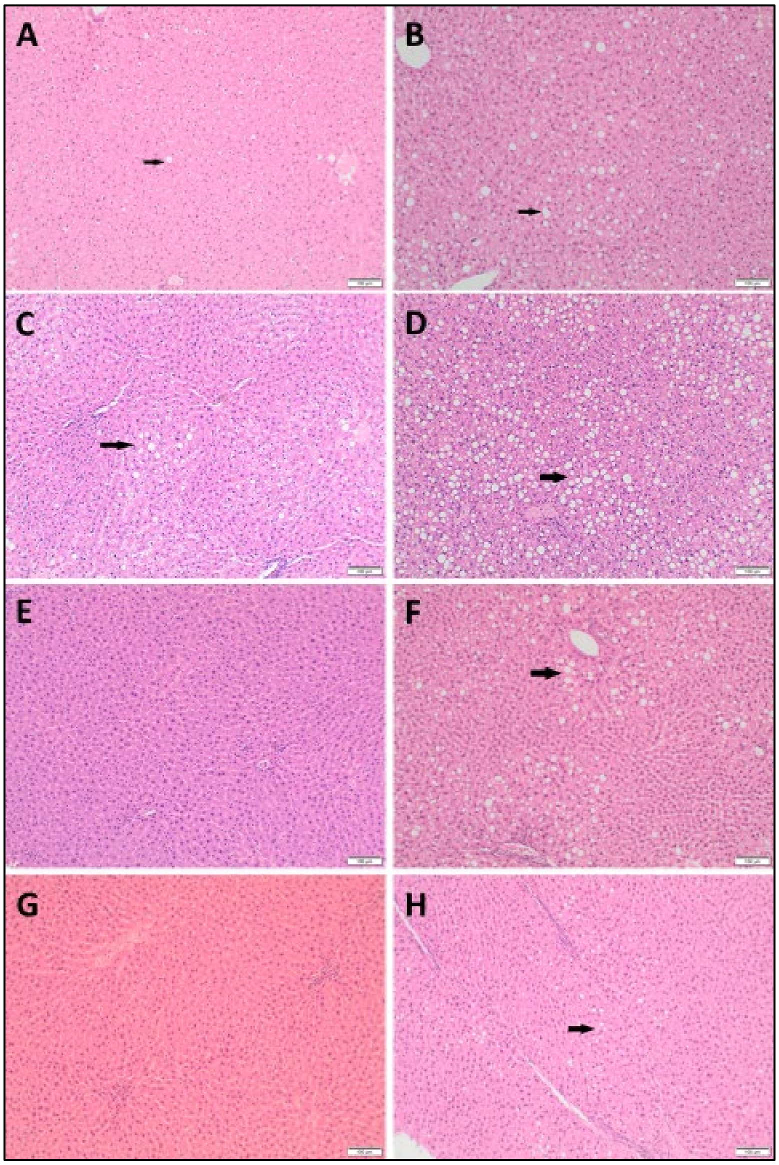

Supplemental Figure S5. Liver steatosis on hematoxylin-eosin-stained liver slices. Arrows on the slices show the lipid accumulation. (**A**) WT male chow – minimal steatosis. (**B**) WT male HFD – moderate steatosis. (**C**) KO male chow – mild steatosis. (**D**) KO male HFD – severe steatosis. **(E)** WT female chow – no steatosis. **(F)** WT female HFD – moderate steatosis **(G)** KO female chow – no steatosis **(H)** KO female HFD – moderate steatosis

HEPATIC TRIGLYCERIDS

We also measured the triglyceride concentration in the liver. For males, a two-way ANOVA detected a significant effect of Diet ($F_{1,20} = 94.66$, $p < 0.0001$; HFD > chow), but the effect of Genotype and Genotype x Diet interaction were not significant. Results were similar for females, with a significant effect of Diet ($F_{1,25} = 24.54$, $p < 0.0001$; HFD > chow), and no significant effect of Genotype or Genotype x Diet interaction. We also compared the concentration of hepatic triglycerides between males and females using a three-way ANOVA, which revealed a significant effect of Sex ($F_{1,45} = 36.03$, $p < 0.0001$; males > females) and Sex x Diet interaction ($F_{1,45} = 27.76$, $p < 0.0001$). *Post hoc* analysis showed that the effect of Sex was only significant in the HFD, but not in the chow-fed group (Supplemental Figure S6).

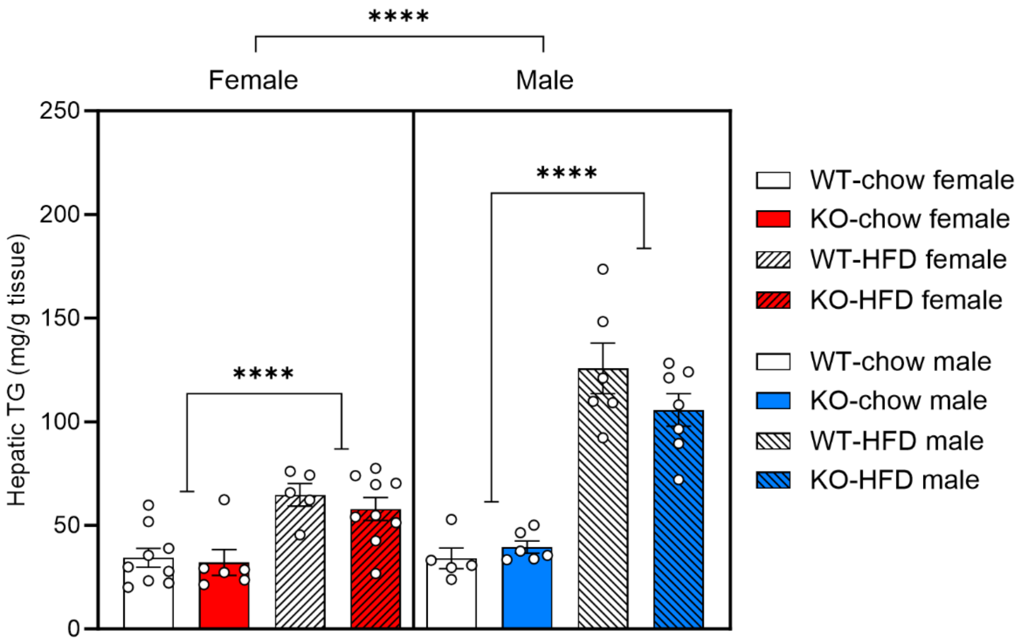

Supplemental Figure S6. Hepatic triglycerides (TG) concentration. The concentration of hepatic triglycerides (mg/g tissue) was higher in HFD compared to chow in both females and males (**** $p < 0.0001$) regardless of Genotype. Males had higher hepatic TG than females (**** $p < 0.0001$), but only with HFD. Female WT-chow: $n =$

9; Female KO-chow: $n = 6$; Female WT-HFD: $n = 5$; Female KO-HFD: $n = 9$; Male WT-chow: $n = 5$; Male KO-chow: $n = 6$; Male WT-HFD: $n = 6$; Male KO-HFD: $n = 7$.

SMALL ANIMAL PET – BRAIN GLUCOSE UPTAKE

In addition to the measurement of FDG uptake in the whole brain, we also conducted a more detailed analysis assessing differences by brain areas.

In WT male rats, HFD decreased (blue) FDG uptake in the nucleus accumbens *(1-2)*, insular cortex *(2-6)*, somatosensory cortex *(2-6)*, piriform cortex *(3)*, cingulum *(4)*, basal amygdaloid nucleus *(4-6)*, striatum *(5)*, regions of the thalamic nucleus *(5-7)*, retrosplenial cortex *(5-7)*, hippocampus *(5-6)*, visual cortex *(7-10)*, dorsal lateral geniculate nucleus *(7)*, periaqueductal grey *(7)*, Edinger-Westphal nucleus *(8-9)*, external cortex of the inferior colliculus *(10)*, and increased (red) FDG uptake only in the dentate gyrus *(9)* compared with chow-fed rats ($p < 0.05$; **Supplemental Figure S7A**). In the case of GHSR-KO male rats, HFD decreased (blue) FDG uptake in the piriform cortex *(1)*, endopiriform nucleus *(2)*, hippocampus *(5-8)*, sensory cortex *(6)*, regions of the thalamic nucleus *(6)*, piriform cortex *(6)*, retrosplenial cortex *(7)*, visual cortex *(8-9)*, and medial lemniscus *(9)*, and increased (red) FDG uptake in the claustrum *(3)*, entorhinal cortex *(9)*, simple and ansiform lobules of the cerebellum *(11-12)* compared with chow-fed rats ($p < 0.05$; **Supplemental Figure S7B**).

In WT female rats, HFD decreased (blue) FDG uptake in the primary motor cortex *(3, 6)*, somatosensory cortex *(4)*, and the hippocampus-CA2 *(7)*, while an increase by HFD was detected in the piriform cortex *(2-3)*, endopiriform nucleus *(2-3)*, accumbens shell *(2)*, stria terminalis *(4)*, insular cortex *(4)*, thalamic nucleus *(6)*, hippocampus (CA1) *(6)*, dorsal nucleus of lateral geniculate body *(6-7)*, entorhinal cortex *(6)*, and superior colliculus *(9)* when compared with chow-fed rats ($p < 0.05$; **Supplemental Figure S7C**). For GHSR-KO female rats, HFD decreased (blue) FDG uptake in the nucleus accumbens shell *(1-2)*, piriform cortex *(2)*, primary motor cortex *(2)*,

somatosensory cortex *(3-6)*, regions of the thalamic nucleus *(4)*, deep gray layer of the superior colliculus *(8-9)*, regions of the pontine reticular nucleus *(9)*, parasubiculum *(9)*, central nucleus of the inferior colliculus *(10)*, dorsal tegmental nucleus *(10)*, principal sensory trigeminal nucleus *(10-11)*, central grey of the pons *(11)*, supragenual nucleus *(11)*, parvicellular part of the medial vestibular nucleus *(11)*, superior vestibular nucleus *(11)*, and increased (red) FDG uptake in the striatum (*3-5*), central amygdala (*5)*, central medial nucleus *(6)*, and dorsal raphe nucleus *(9)*, cuneate nucleus *(12)*, nucleus of the solitary tract *(12)* compared with chow-fed rats ($p < 0.05$; **Supplemental Figure S7D**).

In chow-fed male rats, GHSR deletion increased FDG uptake (red) in the motor cortex *(1)*, orbital cortex *(1)*, piriform cortex *(2-5)*, endopiriform nucleus *(2)*, nucleus accumbens shell *(2)*, primary motor cortex *(3)*, cingulum *(3-4)*, islands of Calleja *(3)*, nucleus accumbens core *(3)*, corpus callosum *(4)*, basal amygdaloid nucleus *(5)*, zona incerta *(5)*, regions of the thalamic nucleus *(6)*, lateral geniculate nuclei *(6)*, auditory cortex *(6)*, hippocampus *(6-8)*, dentate gyrus *(7-8)*, medial geniculate nuclei *(8)*, ventral tegmental area *(8)*, microcellular tegmental nucleus *(9)*, entorhinal cortex *(9)*, ectorhinal cortex *(9)* and inferior colliculus *(10)* compared with WT rats ($p < 0.05$; **Supplemental Figure S7E**). For HFD-fed male rats, GHSR deletion increased FDG uptake (red) in the motor cortex (1), orbital cortex *(1)*, nucleus accumbens *(2)*, cingulum *(2-5)*, somatosensory cortex *(2-4)*, piriform cortex *(3-8)*, endopiriform nucleus *(3)*, ventral pallidum *(3-4)*, striatum *(3-5)*, amygdala *(4-5)*, basal amygdaloid nucleus *(4-5)*, regions of the thalamic nucleus *(5-7)*, auditory cortex *(5-6)*, habenula *(6)*, retrosplenial granular cortex *(8)*, deep mesencephalic nucelus *(8),* entorhinal cortex *(9)*, visual cortex *(9)* compared with WT rats ($p < 0.05$; **Supplemental Figure S7F**).

In chow-fed female rats, GHSR deletion increased (red) FDG uptake in the striatum *(1)*, endopiriform nucleus *(2)*, somatosensory cortex *(3-6)*, bed nucleus of stria terminalis *(3-4)*, stria terminalis *(4)*, auditory cortex *(6-9)*, regions of the thalamic nucleus *(6,8,9)*, hippocampus *(7-8)*, posteromedial cortical amygdaloid nucleus *(7)*, medial geniculate nuclei *(8)*, ventral tegmental area *(8)*, visual cortex *(9)*, ectorhinal cortex *(9)*, parasubiculum *(9)*, microcellular tegmental nucleus *(9)*, external cortex of the inferior colliculus *(10)*, external cuneate nucleus *(12)*, and decreased (blue) FDG uptake in the simple lobule of cerebellum *(11)* compared with WT rats ($p < 0.05$; **Supplemental Figure S7G**). For female rats fed HFD, GHSR deletion increased (red) FDG uptake in the primary motor cortex *(1-3)*, striatum *(1,3)*, lateral septal nucleus *(2-3)*, bed nucleus of the stria terminalis *(3-4)*, somatosensory cortex *(3-6)*, lateral hypothalamic area *(7)*, retrosplenial granular cortex *(7)*, deep mesencephalic nucleus *(8)*, medial geniculate nucleus *(8)*, subiculum *(9)*, nucleus of the solitary tract *(12)*, regions of the reticular nucleus *(12)*, and decreased (blue) FDG uptake in the anterior olfactory nucleus *(1)*, dorsal tenia tecta *(1)*, piriform cortex *(2)*, fornix *(4)*, stria medullaris *(4)*, stria terminalis *(4)*, internal capsule *(4)*, dentate gyrus *(7)*, compared with WT rats ($p < 0.05$; **Supplemental Figure S7H**).

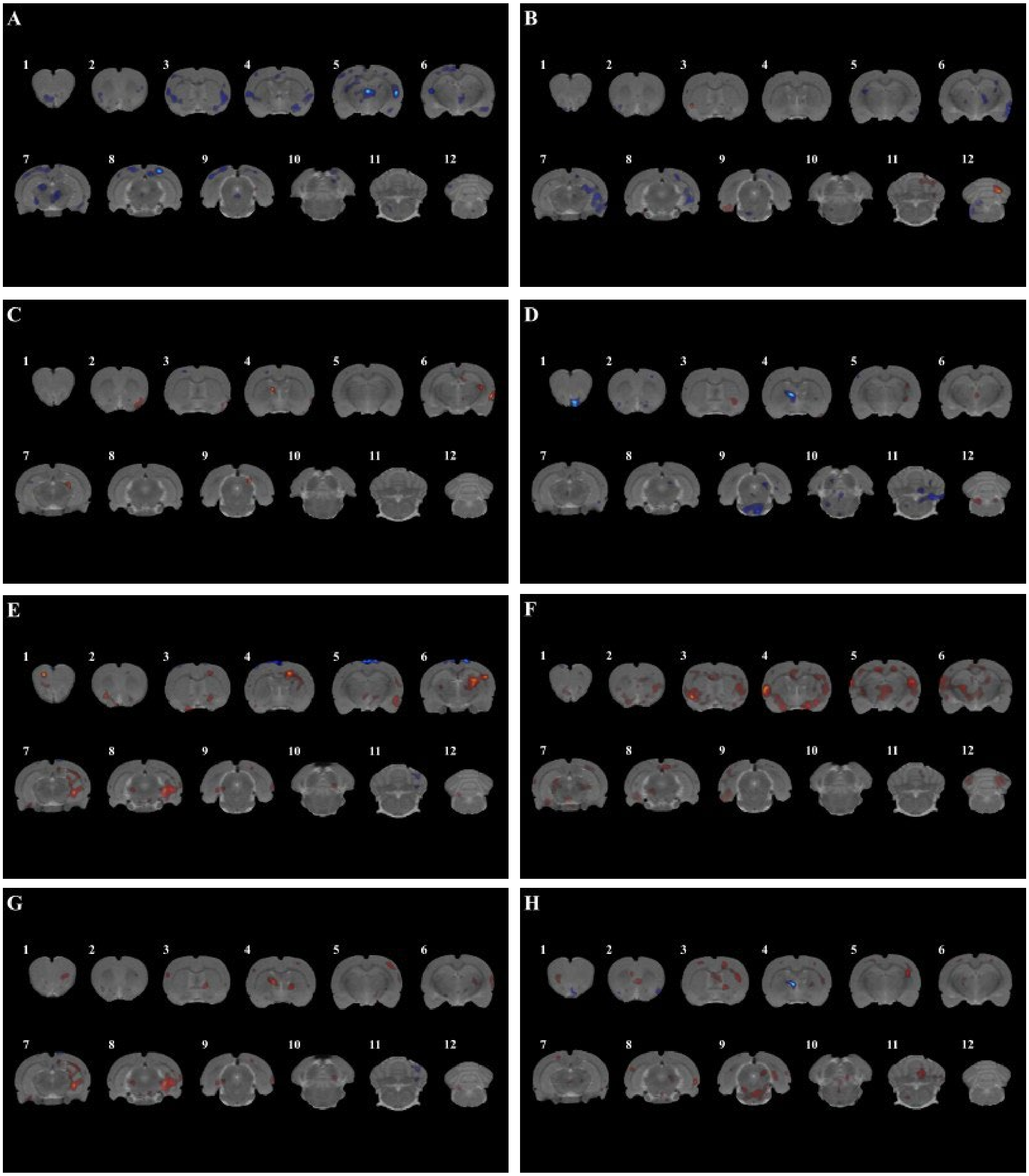

Supplemental Figure S7. Small animal PET. (**A-B**) Statistical parametric maps ($p < 0.05$) of [^{18}F] fluorodeoxyglucose (FDG) uptake as a function of Diet (n = 6/treatment group) in WT **(A)** and GHSR-KO **(B)** male rats. HFD increased (red) and decreased (blue) FDG uptake in different brain regions. (**C-D**) Statistical parametric maps ($p < 0.05$) of FDG uptake as a function of Diet (n = 6/treatment group) in WT **(C)** and GHSR-KO **(D)** female

rats. HFD increased (red) and decreased (blue) FDG uptake in different brain regions. (**E-F**) Statistical parametric maps ($p < 0.05$) of FDG uptake as a function of Genotype ($n = 6$/treatment group) in Chow **(E)** and HFD **(F)** fed male rats. GHSR deletion increased (red) and decreased (blue) FDG uptake in different brain regions. (**G-H**) Statistical parametric maps ($p < 0.05$) of FDG uptake as a function of Genotype ($n = 6$/treatment group) in Chow **(G)** and HFD **(H)** fed female rats. GHSR deletion increased (red) and decreased (blue) FDG uptake in different brain regions.

ENDOCRINE ASSAYS

Glucose Tolerance Test

A three-way repeated-measures (RM) ANOVA (Genotype x Diet x Time) only revealed a significant Time effect ($F_{1.59, 38.15} = 26.31$, $p < 0.001$) for glucose levels in male rats. Two-way RM ANOVAs in each diet group (Genotype x Time) also revealed only a Time effect (Chow: $F_{1.347, 14.81} = 18.37$, $p < 0.001$; HFD: $F_{1.530, 19.88} = 13.60$, $p < 0.001$). *Post hoc* comparisons indicated that blood glucose levels were higher at all timepoints compared with baseline in chow-fed and HFD animals ($p < 0.01$; **Supplemental Figure S8A**). Glucose levels at 120 min were lower than levels at 20 min ($p < 0.05$) in chow-fed animals, but not in HFD-fed (**Supplemental Figure S8A**). There were no significant main effects or interactions on insulin levels (**Supplemental Figure S8B**) in male rats.

In female rats, the three-way RM ANOVA also revealed a significant Time effect ($F_{1.644, 46.04} = 18.757$, $p < 0.0001$) for glucose levels. Two-way RM ANOVAs in each diet group (Genotype x Time) also indicated Time effect only (Chow: $F_{1.157, 17.35} = 12.04$, $p < 0.01$; HFD: $F_{2.015, 26.19} = 8.259$, $p < 0.01$). *Post hoc* comparisons indicated that blood glucose levels were higher at all time points compared with baseline in the chow-fed animals ($p < 0.05$), but only at 20 ($p < 0.05$) and 60 min ($p < 0.01$) in HFD-fed (**Supplemental Figure S8C**). Glucose levels at 120 min were lower than levels at 20 min ($p < 0.05$) in both diets (**Supplemental Figure S8C**). For insulin

levels in female rats, the ANOVA also indicated a significant effect of Time ($F_{1, 28} = 25.65$, $p <$ 0.0001; **Supplemental Figure S8D**), indicating that plasma insulin levels were higher at 60 min than at baseline, regardless of genotype.

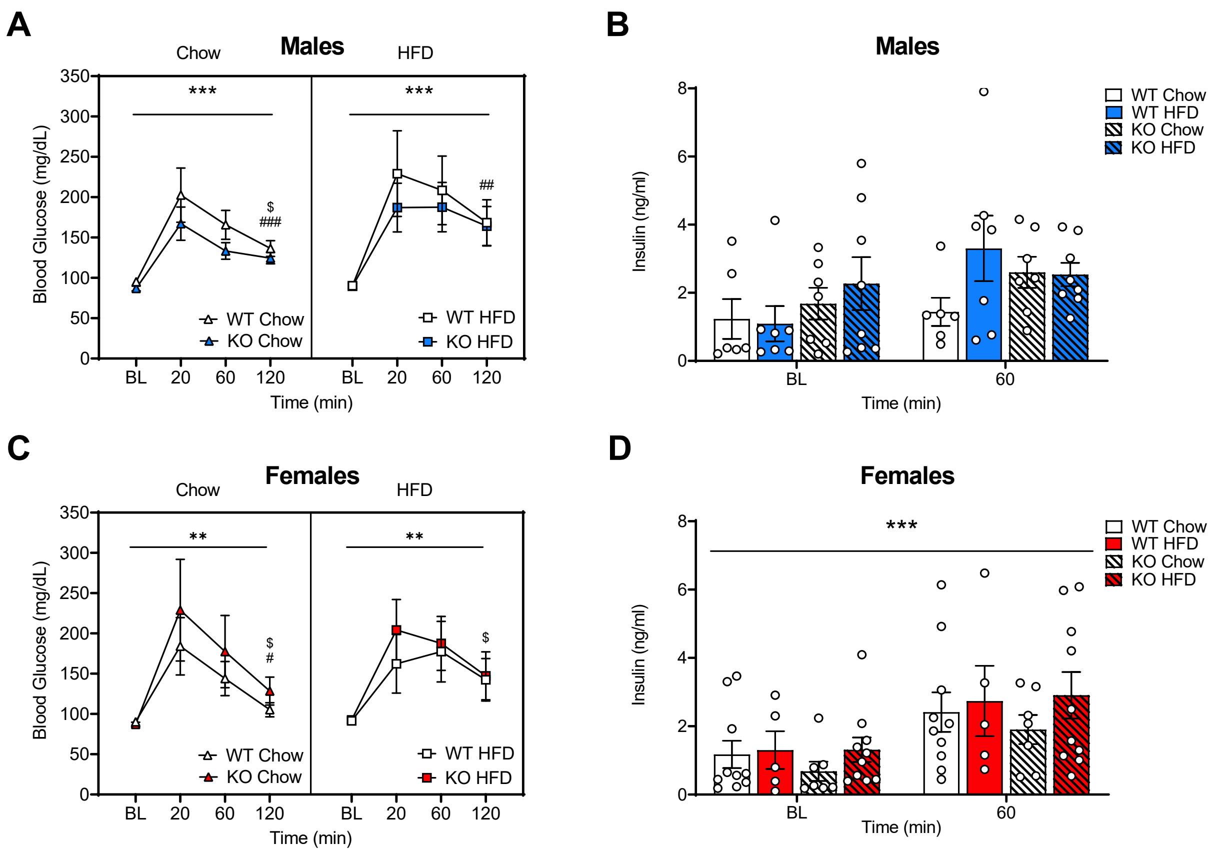

Supplemental Figure S8. Increased blood glucose levels in the glucose tolerance test in male and female rats, regardless of genotype. (**A**) Time had a significant effect on blood glucose levels in both diets, regardless of genotype ($^{***}p < 0.001$). At 120 min, blood glucose levels of male rats, on chow or HFD, were higher than blood glucose levels at baseline ($^{###}p < 0.001$, $^{##}p < 0.01$), and lower than at 20 min in the chow-fed only ($^{\$}p < 0.05$). (**B**) There was no significant difference between WT and GHSR-KO male rats fed chow or HFD in plasma insulin levels during glucose tolerance test. WT: $n = 6$ Chow, $n = 7$ HFD. GHSR-KO: $n = 7$ Chow, $n = 8$ HFD. (**C**) Time had a significant effect on blood glucose levels across all female groups ($^{**}p < 0.01$). At 120 min, blood glucose levels of female rats were lower than blood glucose levels at 20 min in both diets ($^{\$}p < 0.05$) and higher than baseline in chow diet only ($^{#}p <$ 0.05). (**D**) Plasma insulin levels at 60 min were higher than at baseline across all female groups ($^{***}p < 0.001$), regardless of genotype. WT: $n = 10$ Chow, $n = 5$ HFD. GHSR-KO: $n = 7$ Chow, $n = 10$ HFD.

Insulin Tolerance Test

A three-way RM ANOVA (Genotype x Diet x Time) indicated a significant Time effect ($F_{2.616, 60.16} = 253.2$, $p < 0.001$) and a significant interaction of Diet x Genotype ($F_{1, 23} = 6.091$, $p < 0.05$) in glucose levels in male rats. Two-way RM ANOVA (Genotype x Time) in chow-fed males indicated a Time effect only ($F_{2.235, 22.35} = 113.4$, $p < 0.0001$). In HFD-fed male rats, two-way RM ANOVA (Genotype x Time) revealed a significant effect of Time ($F_{2.555, 33.21} = 144.9$, $p < 0.0001$), and Genotype ($F_{1, 13} = 6.640$, $p < 0.05$; KO > WT). *Post hoc* analyses following the main effect of Time indicated that blood glucose levels at all timepoints were lower than baseline levels, in both diet groups ($p < 0.001$; **Supplemental Figure S9A**).

For females, the three-way RM ANOVA revealed a significant Time effect ($F_{1.859, 53.90} = 92.94$, $p < 0.0001$) for blood glucose levels, which were lower than baseline levels at all timepoints in chow-fed animals, and after 60 min in HFD-fed females, indicated by diet-specific two-way RM ANOVAs (**Supplemental Figure S9B**).

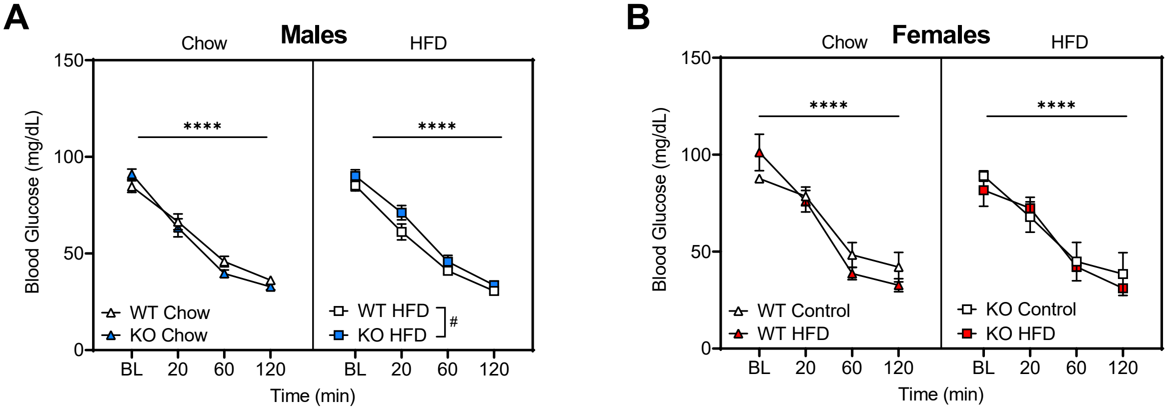

Supplemental Figure S9. Decreased blood glucose levels in the insulin tolerance test in male and female rats. (**A**) Blood glucose levels at all timepoints decreased from baseline across all male groups ($^{****}p < 0.0001$). Blood glucose levels in GHSR-KO HFD-fed male rats were higher when compared to WT HFD-fed male rats ($^{\#}p < 0.05$). (**B**) Blood glucose levels at all timepoints decreased from baseline across all female groups ($^{****}p < 0.0001$).

Terminal Hormone Analysis

For males, a two-way ANOVAs did not reveal significant main effects or interactions in plasma concentrations of ghrelin, desacyl-ghrelin, LEAP2, corticosterone, insulin, GH, or testosterone at the terminal hormone analysis (after 52 weeks of *ad libitum* access to HFD or chow diet; **Supplemental Figure S10A-F**). A two-way ANOVA showed a significant Genotype x Diet interaction ($F_{1, 24} = 9.814$, $p < 0.01$) for progesterone concentrations. *Post hoc* test indicated increased levels of progesterone in GHSR-KO chow-fed male rats compared to the WT chow-fed ($p < 0.01$) and GHSR-KO HFD-fed groups ($p < 0.05$; **Supplemental Figure S10G**). A two-way ANOVA indicated a main effect of diet ($F_{1, 24} = 7.592$, $p < 0.01$; chow > HFD) for aldosterone levels (**Supplemental Figure S10I**), while we only observed a trend for a main effect of genotype ($F_{1, 24} = 2.772$, $p = 0.109$) with higher aldosterone in GHSR-KO animals compared to WT. Of note, the increased aldosterone levels are consistent with previous data from our lab; in fact, in the originally GHSR-KO cohort, whose characterization was reported in *Zallar et al*[13], we also found that aldosterone was significantly higher in GHSR-KO rats (2094.32 ± 280.79 pg/ml) than in the WT rats (1231.36 ± 152.13 pg/ml; $p < 0.05$; *unpublished data*). The ANOVA indicated a main effect of diet ($F_{1, 24} = 7.852$, $p < 0.01$) for leptin levels, with HFD-fed male rats showing higher levels of leptin compared with chow-fed male rats ($p < 0.05$; **Supplemental Figure S10J**).

For females, two-way ANOVAs did not reveal significant main effects or interactions for plasma concentrations of ghrelin, desacyl-ghrelin, corticosterone, testosterone, insulin, LEAP2 or GH at the time of terminal hormone analysis (*p's* > 0.05).

We were interested in the previously described[14] differences of the ghrelin system between males and females, in relation to diet and genotype. When we compared hormones of the ghrelin system, and GH between males and females, a three-way ANOVA (Diet x Genotype x Sex) described a significant effect of Sex on LEAP2 level ($p < 0.0001$). Males had higher LEAP2 levels

(Supplemental Figure S11D), while we did not detect significant main effects or interactions for ghrelin, desacyl-ghrelin, or GH levels **(Supplemental Figure S11A-C)**.

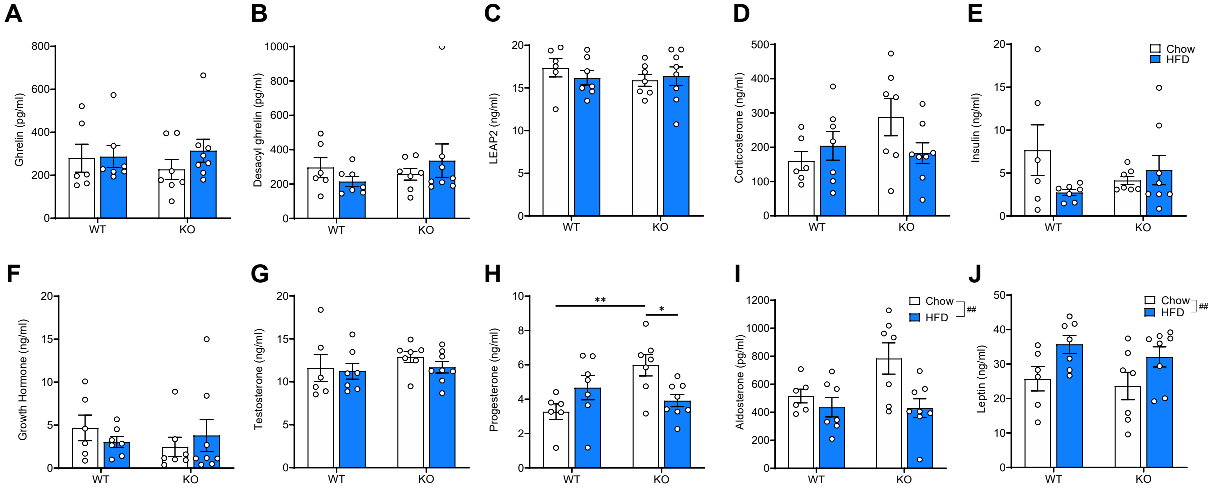

Supplemental Figure S10. Terminal hormone analysis - males. (**A-G**) There was no significant difference between WT and GHSR-KO male rats fed chow or HFD on plasma concentrations of ghrelin, desacyl-ghrelin, LEAP2, corticosterone, insulin, GH, or testosterone. (**H**) GHSR-KO chow-fed male rats had higher levels of progesterone compared with WT chow-fed ($^{**}p < 0.01$) and GHSR-KO HFD-fed groups ($^{*}p < 0.05$). (**I**) Chow-fed male rats had higher levels of aldosterone compared with HFD-fed rats ($^{\#\#}p < 0.01$). (**J**) Leptin levels were significantly higher in HFD-fed male rats compared with chow-fed rats ($^{\#\#}p < 0.01$). WT: n = 6 Chow, n = 7 HFD. GHSR-KO: n = 7 Chow, n = 8 HFD. All data are expressed as mean ± SEM, circles represent each individual rat.

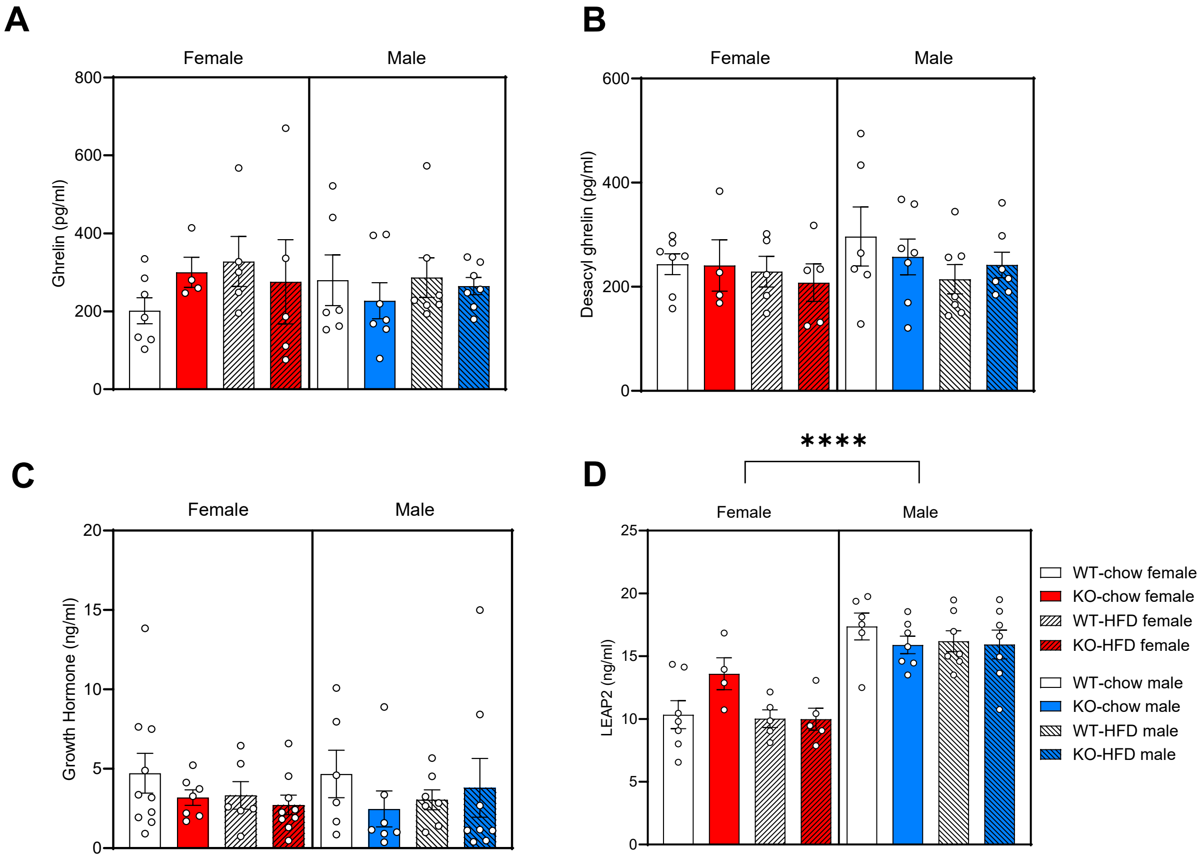

Supplemental Figure S11. Terminal hormone analysis – ghrelin system and GH in males and females. (**A-C**) There was no significant difference regarding Sex, Diet, or Genotype in plasma concentrations of ghrelin, desacyl-ghrelin, or GH. (**D**) Males had higher LEAP2 levels compared to females ($^{****}p < 0.0001$). Males WT: *n* = 6 Chow, *n* = 7 HFD. Females WT: *n* = 7 (GH: *n* = 10) Chow, *n* = 5 (GH: *n* = 6) HFD. Males GHSR-KO: *n* = 7 Chow, *n* = 7 (GH: *n* = 8) HFD. Females GHSR-KO: *n* = 4 (GH: *n* = 7) Chow, *n* = 5 (GH: *n* = 9) HFD. All data are expressed as mean ± SEM, circles represent each individual rat.

GENE EXPRESSION AND PATHWAY ANALYSIS BY RNA-SEQ

Comparisons of BAT gene expression in GHSR-KO and WT on the HFD or regular diet by GSEA are outlined in the **Supplementary Data 2-6**. The differential gene expression patterns in BAT are displayed in the **Supplemental Figure S12**, which complements **Figure 5**.

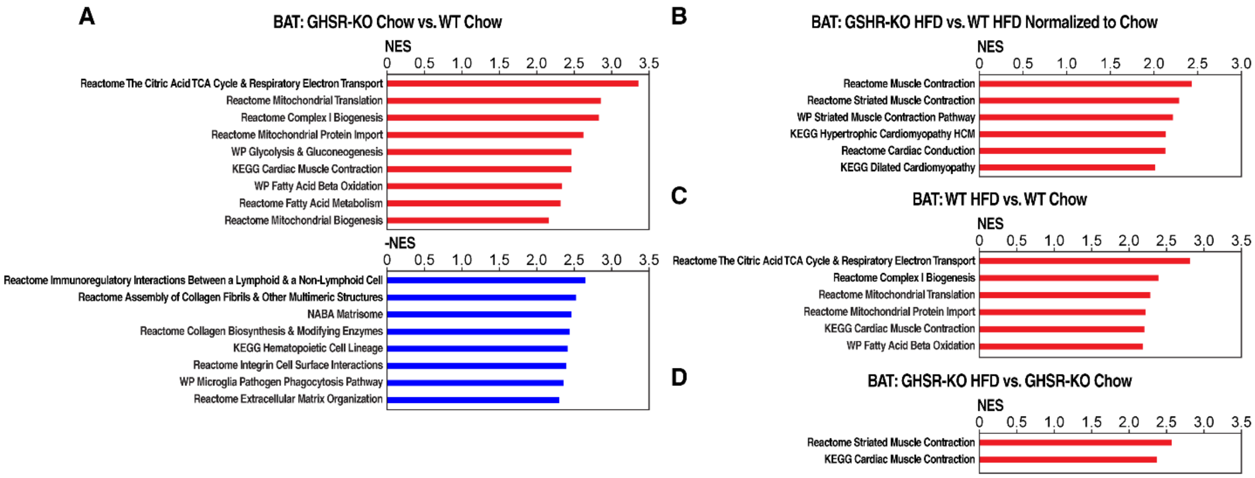

Supplemental Figure S12. Gene Set Enrichment Analysis (GSEA) pathway analysis of brown adipose tissue (BAT). (**A**) GSEA pathway analysis of the BAT of GHSR-KO rats on chow vs. WT rats on chow. Results indicate increased expression of TCA cycle and respiratory electron transport genes; fat and glucose metabolism genes, and BAT skeletal muscle-like gene program in GHSR-KO rats on chow vs. WT rats on chow and increased expression of extracellular matrix, integrins and collagen biosynthesis and modifying enzymes genes in WT rats. (**B**) HFD-induced greater upregulation of genesets representative of skeletal muscle-like genes in the BAT of GHSR-KO vs. WT rats is evident when gene expression in HFD-fed rats is normalized to gene expression in chow-fed rats. (**C**) Representative genesets differentially regulated in the BAT of WT rats on HFD vs. WT rats on chow suggest higher expression of TCA cycle and respiratory electron transport; fat metabolism gene, and muscle like charge transport pathways with the HFD. (**D**) For BAT, representative genesets differentially regulated in GHSR-KO rats on HFD vs. GHSR-KO on chow indicate that HFD primarily induced skeletal muscle-like genes in GHSR-KO rats. (Genesets shown have FWER p-values < 0.05; overlapping genesets not shown; complete list is in Supplemental Tables S2-5).

LIPIDOMIC ANALYSIS BY MASS SPECTROMETRY

Analysis of lipid extracts of adipose tissue by mass spectrometry resulted in the assignment of 43 TG species. A total of 79 samples were analyzed consisting of 4 groups (WT chow, WT HFD, GHSR-KO chow, GHSR-KO HFD) for each sex and for each type of adipose tissue (BAT, WAT). All the groups consisted of an $n = 5$, except for female GHSR-KO chow WAT that had an $n = 4$. A two-way (Genotype x Diet) ANOVA was used, followed by Tukey's multiple comparison tests to assess group differences for males and females and for BAT and WAT samples. These results are showed in **Supplemental Table 2** (WAT) and **Supplemental Table 3** (BAT). Genotype affected the TG profile of adipose tissue, but was highly localized for WAT samples in males, and was only observed for animals fed a chow diet. In every case of significance, an increase in the TG species was observed for GHSR-KO rats compared to WT rats. In WAT samples, 11 TG

species (48:4, 52:4, 52:3, 54:7,54:6, 54:5, 58:5, 58:4, 60:7, 60:6, 60:5) were significantly increased in GHSR-KO males compared to WT males fed a chow diet. In BAT samples, 2 TG species (58:5 and 58:4) were significantly increased in GHSR-KO males compared with WT males fed with a chow diet, whereas in females fed with a chow diet only TG 50:4 was significantly increased in GHSR-KO compared with WT.

Diet greatly affected the TG profile of adipose tissue regardless of sex, genotype, and type of adipose tissue (See **Supplemental Table 2** and **Supplemental Table 3**). In general, for a TG series with the same total number of carbons in the acyl chains, HFD leads to a higher number of TG species with 1-3 carbon double bonds (increase in saturation) and significant decrease in TG species with 3 < carbon double bonds (decrease in unsaturation). This trend was seen across all groups for a series of TG species (TG 52:4.TG 52:3, TG 52:2, **Supplemental Figure S13A** in male rats and **Supplemental Figure S13B** in female rats).

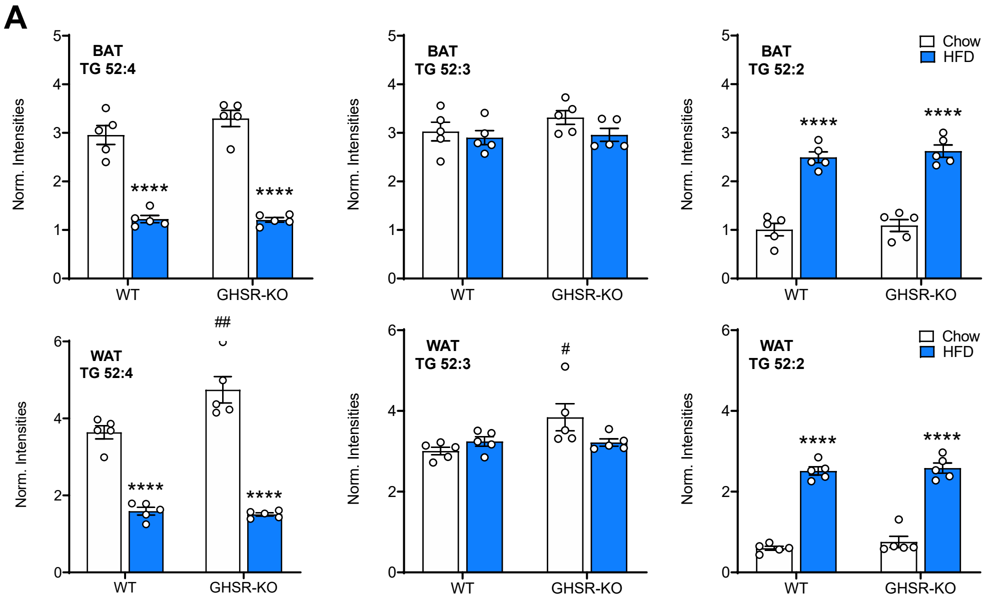

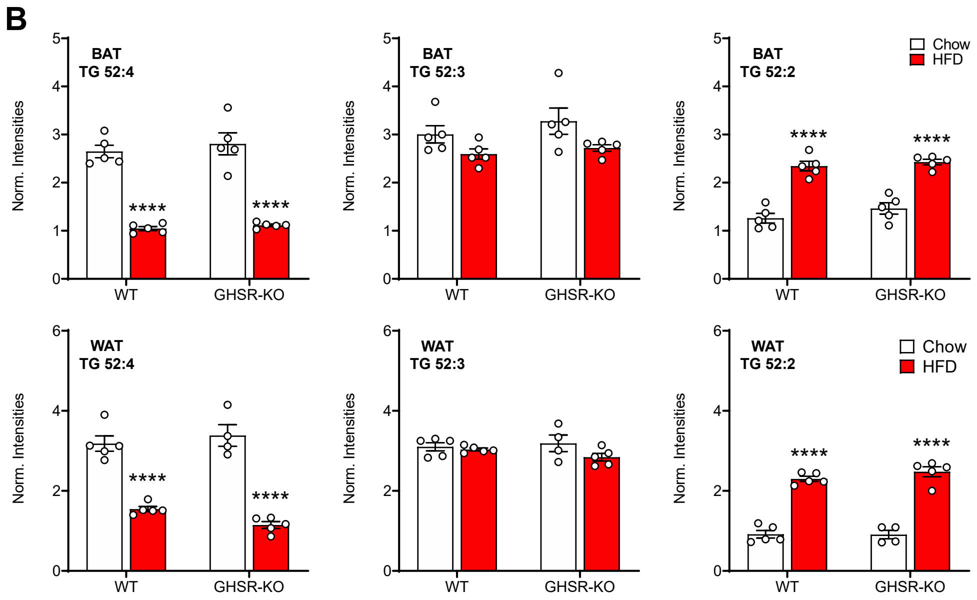

Supplemental Figure S13. **Diet significantly changes TG species in adipose tissue based upon the level of saturation (number of carbon double bonds).** Comparison of TG levels for TG 52:4, 52:3, and 52:2 from rats fed either a Chow or HFD for BAT and WAT for (**A**) males and (**B**) female rats. TG 52:4 was significantly decreased in animals fed HFD, TG 52:2 was significantly increased in animals fed HFD ($^{****}p < 0.0001$). TG 52:4 and 52:3 in WAT samples for GHSR-KO male rats fed a Chow diet was significantly increased compared to WT male rats fed a Chow diet ($^{\#\#}p < 0.01$, $^{\#}p < 0.05$).

Supplemental Table 2. TG Species for WAT samples

Species	Males				Females			
	WT Chow (5)	WT HFD (5)	KO Chow (5)	KO HFD (5)	WT Chow (5)	WT HFD (5)	KO Chow (4)	KO HFD (5)
TG 40:2	0.006 ± 0.001	0.012 ± 0.001 (*)	0.009 ± 0.001	0.011 ± 0.001	0.005 ± 0.001	0.012 ± 0.002	0.010 ± 0.003	0.008 ± 0.002
TG 44:2	0.011 ± 0.005	0.008 ± 0.001	0.018 ± 0.009	0.007 ± 0.0004	0.006 ± 0.001	0.012 ± 0.002	0.013 ± 0.007	0.008 ± 0.001
TG 46:3	0.024 ± 0.008	0.016 ± 0.001	0.034 ± 0.011	0.013 ± 0.001	0.013 ± 0.004	0.019 ± 0.002	0.020 ± 0.007	0.012 ± 0.001
TG 46:2	0.026 ± 0.005	0.029 ± 0.003	0.043 ± 0.007	0.024 ± 0.002	0.025 ± 0.008	0.034 ± 0.002	0.029 ± 0.008	0.024 ± 0.002
TG 46:1	0.016 ± 0.004	0.026 ± 0.003	0.026 ± 0.005	0.021 ± 0.001	0.018 ± 0.006	0.032 ± 0.002	0.024 ± 0.006	0.022 ± 0.002
TG 46:0	0.009 ± 0.003	0.012 ± 0.002	0.014 ± 0.003	0.010 ± 0.001	0.013 ± 0.002	0.016 ± 0.003	0.014 ± 0.002	0.013 ± 0.001
TG 48:4	0.041 ± 0.007	0.015 ± 0.002 (**)	0.061 ± 0.005 (*)	0.012 ± 0.002 (****)	0.033 ± 0.012	0.017 ± 0.001	0.038 ± 0.009	0.011 ± 0.002
TG 48:3	0.113 ± 0.021	0.076 ± 0.009	0.159 ± 0.012	0.062 ± 0.006 (***)	0.106 ± 0.044	0.083 ± 0.008	0.114 ± 0.022	0.048 ± 0.006
TG 48:2	0.185 ± 0.028	0.185 ± 0.018	0.265 ± 0.032	0.152 ± 0.008 (*)	0.203 ± 0.049	0.200 ± 0.012	0.224 ± 0.038	0.137 ± 0.009
TG 48:1	0.112 ± 0.019	0.179 ± 0.018	0.162 ± 0.029	0.158 ± 0.005	0.127 ± 0.028	0.201 ± 0.010	0.152 ± 0.022	0.153 ± 0.006
TG 48:0	0.045 ± 0.009	0.056 ± 0.009	0.063 ± 0.016	0.056 ± 0.007	0.066 ± 0.008	0.072 ± 0.012	0.072 ± 0.011	0.075 ± 0.006
TG 50:5	0.088 ± 0.014	0.019 ± 0.003 (****)	0.118 ± 0.007	0.014 ± 0.001 (****)	0.074 ± 0.026	0.022 ± 0.002	0.081 ± 0.013	0.011 ± 0.002 (*)
TG 50:4	0.459 ± 0.066	0.180 ± 0.022 (***)	0.611 ± 0.039	0.149 ± 0.014 (****)	0.396 ± 0.123	0.190 ± 0.020	0.416 ± 0.053	0.109 ± 0.016 (*)

TG 50:3	0.930 ± 0.127	0.688 ± 0.060	1.242 ± 0.109	0.600 ± 0.032 (***)	0.866 ± 0.235	0.692 ± 0.048	0.980 ± 0.124	0.444 ± 0.044
TG 50:2	1.004 ± 0.105	1.151 ± 0.075	1.370 ± 0.206	1.064 ± 0.026	1.191 ± 0.078	1.192 ± 0.027	1.275 ± 0.146	1.001 ± 0.034
TG 50:1	0.248 ± 0.022	0.639 ± 0.071 (***)	0.334 ± 0.053	0.648 ± 0.058 (**)	0.340 ± 0.039	0.711 ± 0.072 (**)	0.346 ± 0.045	0.785 ± 0.058 (***)
TG 52:6	0.215 ± 0.033	0.027 ± 0.003 (****)	0.284 ± 0.017	0.022 ± 0.002 (****)	0.157 ± 0.049	0.033 ± 0.002 (*)	0.178 ± 0.027	0.015 ± 0.004 (**)
TG 52:5	1.388 ± 0.177	0.336 ± 0.032 (****)	1.790 ± 0.098	0.295 ± 0.018 (****)	1.025 ± 0.226	0.344 ± 0.024 (*)	1.164 ± 0.137	0.198 ± 0.031 (***)
TG 52:4	3.638 ± 0.171	1.590 ± 0.100 (****)	4.744 ± 0.342 (**)	1.506 ± 0.042 (****)	3.182 ± 0.191	1.544 ± 0.065 (****)	3.385 ± 0.272	1.148 ± 0.086 (****)
TG 52:3	3.008 ± 0.094	3.244 ± 0.117	3.842 ± 0.336 (*)	3.218 ± 0.090	3.100 ± 0.103	3.034 ± 0.037	3.188 ± 0.207	2.842 ± 0.095
TG 52:2	0.598 ± 0.050	2.514 ± 0.100 (****)	0.755 ± 0.139	2.584 ± 0.126 (****)	0.915 ± 0.094	2.300 ± 0.064 (****)	0.908 ± 0.105	2.478 ± 0.124 (****)
TG 54:7	0.528 ± 0.042	0.041 ± 0.005 (****)	0.714 ± 0.057 (**)	0.037 ± 0.002 (****)	0.383 ± 0.046	0.052 ± 0.004 (****)	0.415 ± 0.059	0.031 ± 0.005 (****)
TG 54:6	2.920 ± 0.136	0.404 ± 0.029 (****)	3.844 ± 0.326 (**)	0.399 ± 0.012 (****)	2.018 ± 0.166	0.415 ± 0.015 (****)	2.175 ± 0.188	0.303 ± 0.052 (****)
TG 54:5	4.362 ± 0.257	1.698 ± 0.081 (****)	5.532 ± 0.464 (*)	1.738 ± 0.052 (****)	3.408 ± 0.178	1.534 ± 0.037 (****)	3.548 ± 0.234	1.348 ± 0.094 (****)
TG 54:4	2.838 ± 0.186	3.320 ± 0.123	3.492 ± 0.318	3.474 ± 0.126	2.584 ± 0.102	2.796 ± 0.097	2.610 ± 0.168	2.772 ± 0.091
TG 54:3	0.524 ± 0.051	2.748 ± 0.092 (****)	0.596 ± 0.082	2.942 ± 0.132 (****)	0.710 ± 0.076	2.228 ± 0.063 (****)	0.643 ± 0.089	2.412 ± 0.089 (****)
TG 56:8	0.192 ± 0.018	0.016 ± 0.003 (****)	0.238 ± 0.036	0.017 ± 0.002 (****)	0.113 ± 0.022	0.027 ± 0.003 (**)	0.129 ± 0.022	0.013 ± 0.004 (***)
TG 56:7	0.268 ± 0.020	0.065 ± 0.010 (****)	0.321 ± 0.041	0.067 ± 0.003 (****)	0.177 ± 0.029	0.093 ± 0.011 (*)	0.202 ± 0.024	0.060 ± 0.010 (**)
TG 56:6	0.209 ± 0.012	0.102 ± 0.015 (**)	0.251 ± 0.031	0.111 ± 0.005 (***)	0.147 ± 0.015	0.137 ± 0.016	0.171 ± 0.017	0.095 ± 0.013 (*)
TG 56:5	0.140 ± 0.008	0.104 ± 0.013	0.180 ± 0.020	0.115 ± 0.005 (*)	0.103 ± 0.007	0.114 ± 0.009	0.115 ± 0.007	0.096 ± 0.010
TG 56:4	0.088 ± 0.004	0.111 ± 0.009	0.115 ± 0.012	0.119 ± 0.005	0.075 ± 0.004	0.100 ± 0.003 (**)	0.077 ± 0.004	0.090 ± 0.006
TG 56:3	0.044 ± 0.001	0.081 ± 0.005 (***)	0.064 ± 0.007	0.089 ± 0.005 (*)	0.048 ± 0.002	0.071 ± 0.002 (***)	0.047 ± 0.004	0.072 ± 0.004 (***)
TG 56:2	0.009 ± 0.0004	0.027 ± 0.003 (***)	0.015 ± 0.002	0.031 ± 0.003 (***)	0.012 ± 0.002	0.026 ± 0.003 (***)	0.012 ± 0.001	0.032 ± 0.002 (****)
TG 58:8	0.072 ± 0.007	0.011 ± 0.003 (****)	0.073 ± 0.009	0.012 ± 0.001 (****)	0.037 ± 0.006	0.017 ± 0.003 (*)	0.042 ± 0.005	0.011 ± 0.002 (**)
TG 58:7	0.054 ± 0.006	0.018 ± 0.004 (**)	0.060 ± 0.008	0.020 ± 0.001 (***)	0.032 ± 0.004	0.023 ± 0.005	0.036 ± 0.005	0.017 ± 0.003 (*)
TG 58:6	0.050 ± 0.004	0.020 ± 0.003 (***)	0.065 ± 0.005	0.021 ± 0.001 (****)	0.034 ± 0.006	0.023 ± 0.003	0.037 ± 0.004	0.016 ± 0.003 (*)
TG 58:5	0.090 ± 0.004	0.038 ± 0.003 (****)	0.130 ± 0.009 (***)	0.037 ± 0.001 (****)	0.074 ± 0.006	0.037 ± 0.003 (****)	0.077 ± 0.004	0.029 ± 0.003 (****)
TG 58:4	0.064 ± 0.002	0.079 ± 0.004	0.091 ± 0.007 (**)	0.083 ± 0.003	0.065 ± 0.001	0.068 ± 0.003	0.065 ± 0.003	0.068 ± 0.003
TG 58:3	0.015 ± 0.0003	0.056 ± 0.004 (****)	0.022 ± 0.002	0.060 ± 0.003 (****)	0.022 ± 0.003	0.047 ± 0.003 (****)	0.018 ± 0.003	0.056 ± 0.002 (****)
TG 60:7	0.055 ± 0.003	0.008 ± 0.001 (****)	0.084 ± 0.008 (**)	0.008 ± 0.0004 (****)	0.036 ± 0.003	0.007 ± 0.0004 (****)	0.038 ± 0.001	0.005 ± 0.001 (****)
TG 60:6	0.079 ± 0.006	0.031 ± 0.002 (***)	0.114 ± 0.012 (**)	0.033 ± 0.001 (****)	0.060 ± 0.004	0.027 ± 0.001 (****)	0.060 ± 0.004	0.023 ± 0.002 (****)
TG 60:5	0.043 ± 0.004	0.062 ± 0.004 (*)	0.061 ± 0.005 (*)	0.070 ± 0.002	0.041 ± 0.002	0.049 ± 0.003	0.039 ± 0.003	0.052 ± 0.002 (*)
TG 60:4	0.010 ± 0.001	0.047 ± 0.002 (****)	0.016 ± 0.002	0.053 ± 0.002 (****)	0.014 ± 0.002	0.036 ± 0.002 (****)	0.011 ± 0.001	0.038 ± 0.002 (****)

Supplemental Table 2. Data are presented as mean ± standard error of the mean (SEM). The number of samples tested are noted in parentheses. Tukey's Multiple Comparisons Test for Groups. [red] = Statistically lower value than chow diet counterparts of the same sex and genotype; [cyan] = Statistically higher value than chow diet counterparts of the same sex and genotype; [yellow] = Statistically higher value than WT counterparts of the same sex and diet; [green] = Statistically lower than WT counterparts of the same sex and diet. *p value*: *< 0.05, **< 0.01, ***< 0.001, ****< 0.0001. WT: wild type; KO: knockout; GHS-R: growth; HFD: high-fat diet paradigm.

Supplemental Table 3. TG Species for BAT samples

Species	Males				Females			
	WT Chow (5)	WT HFD (5)	KO Chow (5)	KO HFD (5)	WT Chow (5)	WT HFD (5)	KO Chow (4)	KO HFD (5)
TG 40:2	0.017 ± 0.003	0.018 ± 0.004	0.021 ± 0.004	0.019 ± 0.003	0.023 ± 0.004	0.021 ± 0.003	0.025 ± 0.003	0.016 ± 0.003
TG 44:2	0.010 ± 0.002	0.006 ± 0.001	0.011 ± 0.002	0.006 ± 0.0008	0.011 ± 0.001	0.007 ± 0.001	0.013 ± 0.001	0.007 ± 0.001 (*)
TG 46:3	0.016 ± 0.004	0.010 ± 0.001	0.018 ± 0.003	0.011 ± 0.001	0.013 ± 0.002	0.010± 0.001	0.017 ± 0.001	0.009 ± 0.0004 (**)
TG 46:2	0.048 ± 0.013	0.026 ± 0.002	0.049 ± 0.014	0.025 ± 0.002	0.048 ± 0.005	0.029 ± 0.004	0.062 ± 0.008	0.029 ± 0.003 (**)
TG 46:1	0.038 ± 0.014	0.024 ± 0.002	0.040 ± 0.014	0.024 ± 0.002	0.038 ± 0.006	0.030 ± 0.005	0.058 ± 0.009	0.028 ± 0.003 (*)
TG 46:0	0.021 ± 0.005	0.017 ± 0.001	0.022 ± 0.005	0.016 ± 0.002	0.034 ± 0.004	0.020 ± 0.002	0.048 ± 0.006	0.021 ± 0.002 (***)
TG 48:4	0.030 ± 0.006	0.010 ± 0.001 (*)	0.035 ± 0.006	0.010 ± 0.001 (**)	0.025 ± 0.002	0.009 ± 0.001 (****)	0.030 ± 0.001	0.010 ± 0.001 (****)
TG 48:3	0.115 ± 0.030	0.053 ± 0.004	0.125 ± 0.034	0.046 ± 0.004	0.103 ± 0.012	0.050 ± 0.006 (**)	0.135 ± 0.012	0.052 ± 0.004 (****)
TG 48:2	0.284 ± 0.064	0.163 ± 0.012	0.303 ± 0.072	0.159 ± 0.016	0.330 ± 0.027	0.181 ± 0.021 (**)	0.429 ± 0.034	0.185 ± 0.019 (****)
TG 48:1	0.162 ± 0.042	0.166 ± 0.007	0.177 ± 0.047	0.165 ± 0.012	0.200 ± 0.025	0.187 ± 0.017	0.283 ± 0.033	0.191 ± 0.015
TG 48:0	0.064 ± 0.009	0.076 ± 0.003	0.070 ± 0.007	0.083 ± 0.005	0.101 ± 0.008	0.071 ± 0.004	0.128 ± 0.011	0.087 ± 0.003 (**)
TG 50:5	0.054 ± 0.009	0.012 ± 0.002 (***)	0.059 ± 0.007	0.012 ± 0.001 (***)	0.047 ± 0.003	0.013 ± 0.001 (****)	0.053 ± 0.003	0.015 ± 0.001 (****)
TG 50:4	0.318 ± 0.046	0.113 ± 0.009 (**)	0.358 ± 0.049	0.105 ± 0.009 (***)	0.294 ± 0.014	0.100 ± 0.008 (****)	0.342 ± 0.015 (*)	0.110 ± 0.005 (****)
TG 50:3	0.759 ± 0.139	0.464 ± 0.033	0.847 ± 0.147	0.438 ± 0.028	0.733 ± 0.070	0.414 ± 0.028 (**)	0.923 ± 0.075	0.430 ± 0.017 (****)
TG 50:2	1.251 ± 0.153	1.016 ± 0.043	1.396 ± 0.151	1.024 ± 0.058	1.482 ± 0.090	1.017 ± 0.049 (**)	1.770 ± 0.112	1.053 ± 0.038 (****)
TG 50:1	0.347 ± 0.044	0.742 ± 0.018 (****)	0.385 ± 0.048	0.795 ± 0.052 (****)	0.521 ± 0.049	0.815± 0.039 (**)	0.676 ± 0.069	0.832 ± 0.025
TG 52:6	0.100 ± 0.011	0.019 ± 0.002 (****)	0.113 ± 0.008	0.017 ± 0.001 (****)	0.076 ± 0.005	0.017 ± 0.001 (****)	0.087 ± 0.008	0.020 ± 0.001 (****)
TG 52:5	0.726 ± 0.077	0.212 ± 0.018 (****)	0.839 ± 0.052	0.196 ± 0.011 (****)	0.616 ± 0.038	0.169 ± 0.008 (****)	0.689 ± 0.070	0.189 ± 0.009 (****)
TG 52:4	2.956 ± 0.194	1.224 ± 0.074 (****)	3.296 ± 0.166	1.206 ± 0.049 (****)	2.648 ± 0.130	1.046 ± 0.041 (****)	2.806 ± 0.229	1.114 ± 0.026 (****)
TG 52:3	3.028 ± 0.192	2.902 ± 0.143	3.316 ± 0.142	2.958 ± 0.134	3.004 ± 0.180	2.594 ± 0.106	3.278 ± 0.273	2.720 ± 0.069
TG 52:2	1.006 ± 0.128	2.494 ± 0.112 (****)	1.091 ± 0.122	2.622 ± 0.128 (****)	1.260 ± 0.100	2.344 ± 0.100 (****)	1.464 ± 0.117	2.428 ± 0.058 (****)
TG 54:7	0.309 ± 0.026	0.034 ± 0.003 (****)	0.354 ± 0.040	0.033 ± 0.002 (****)	0.231 ± 0.014	0.030 ± 0.001 (****)	0.242 ± 0.023	0.035 ± 0.001 (****)
TG 54:6	1.838 ± 0.157	0.350 ± 0.021 (****)	2.138 ± 0.277	0.355 ± 0.010 (****)	1.284 ± 0.076	0.293 ± 0.014 (****)	1.288 ± 0.141	0.320 ± 0.009 (****)
TG 54:5	3.024 ± 0.204	1.430 ± 0.112 (***)	3.456 ± 0.376	1.418 ± 0.046 (****)	2.292 ± 0.141	1.111 ± 0.055 (***)	2.332 ± 0.304	1.191 ± 0.051 (**)

TG 54:4	2.318 ± 0.111	2.828 ± 0.221	2.574 ± 0.190	2.848 ± 0.107	2.000 ± 0.136	2.224 ± 0.121	2.038 ± 0.271	2.370 ± 0.118
TG 54:3	0.741 ± 0.047	2.476 ± 0.186 (****)	0.806 ± 0.045	2.584 ± 0.107 (****)	0.778 ± 0.044	2.076 ± 0.113 (****)	0.796 ± 0.080	2.158 ± 0.086 (****)
TG 56:8	0.144 ± 0.015	0.026 ± 0.004 (****)	0.147 ± 0.021	0.026 ± 0.003 (****)	0.098 ± 0.008	0.024 ± 0.001 (****)	0.079 ± 0.012	0.031 ± 0.002 (**)
TG 56:7	0.223 ± 0.019	0.096 ± 0.014 (**)	0.226 ± 0.028	0.098 ± 0.011 (**)	0.165 ± 0.013	0.094 ± 0.004 (**)	0.143 ± 0.021	0.116 ± 0.006
TG 56:6	0.183 ± 0.012	0.144 ± 0.016	0.193 ± 0.019	0.151 ± 0.016	0.159 ± 0.013	0.145 ± 0.008	0.133 ± 0.019	0.175 ± 0.008
TG 56:5	0.123 ± 0.007	0.133 ± 0.013	0.142 ± 0.010	0.142 ± 0.014	0.105 ± 0.007	0.131 ± 0.008	0.098 ± 0.011	0.149 ± 0.006 (**)
TG 56:4	0.090 ± 0.006	0.127 ± 0.010 (*)	0.110 ± 0.004	0.133 ± 0.008	0.072 ± 0.004	0.108 ± 0.008 (**)	0.084 ± 0.006	0.120 ± 0.003 (**)
TG 56:3	0.070 ± 0.007	0.105 ± 0.007 (**)	0.087 ± 0.006	0.114 ± 0.007	0.067 ± 0.006	0.090 ± 0.008	0.084 ± 0.006	0.098 ± 0.002
TG 56:2	0.019 ± 0.002	0.048 ± 0.003(****)	0.025 ± 0.003	0.055 ± 0.005 (****)	0.022 ± 0.003	0.042 ± 0.003 (***)	0.030 ± 0.003	0.044 ± 0.002 (*)
TG 58:8	0.057 ± 0.007	0.021 ± 0.004 (**)	0.056 ± 0.010	0.020 ± 0.003 (**)	0.036 ± 0.005	0.019 ± 0.001 (*)	0.026 ± 0.004	0.026 ± 0.002
TG 58:7	0.048 ± 0.006	0.033 ± 0.007	0.049 ± 0.008	0.034 ± 0.006	0.034 ± 0.004	0.033 ± 0.003	0.026 ± 0.004	0.042 ± 0.003 (*)
TG 58:6	0.044 ± 0.003	0.031 ± 0.005	0.052 ± 0.004	0.032 ± 0.003 (*)	0.031 ± 0.003	0.031 ± 0.002	0.030 ± 0.003	0.037 ± 0.002 (*)
TG 58:5	0.107 ± 0.007	0.049 ± 0.004 (****)	0.135 ± 0.010 (*)	0.050 ± 0.003 (****)	0.074 ± 0.006	0.036 ± 0.002 (**)	0.089 ± 0.010	0.041 ± 0.001 (***)
TG 58:4	0.095 ± 0.006	0.106 ± 0.009	0.122 ± 0.004 (*)	0.110 ± 0.006	0.073 ± 0.008	0.073 ± 0.005	0.093 ± 0.010	0.084 ± 0.004
TG 58:3	0.030 ± 0.003	0.085 ± 0.006(****)	0.039 ± 0.003	0.094 ± 0.006 (****)	0.029 ± 0.004	0.063 ± 0.004 (****)	0.040 ± 0.003	0.068 ± 0.003 (***)
TG 60:7	0.051 ± 0.006	0.010 ± 0.001 (**)	0.072 ± 0.014	0.011 ± 0.0004 (***)	0.026 ± 0.001	0.007 ± 0.001 (****)	0.031 ± 0.004	0.009±0.0003(****)
TG 60:6	0.082 ± 0.004	0.040 ± 0.005 (*)	0.106 ± 0.015	0.040 ± 0.002 (***)	0.043 ± 0.003	0.024 ± 0.002	0.054 ± 0.009	0.028 ± 0.002 (**)
TG 60:5	0.056 ± 0.004	0.080 ± 0.009	0.070 ± 0.007	0.083 ± 0.004	0.035 ± 0.004	0.049 ± 0.004	0.044 ± 0.007	0.055 ± 0.005
TG 60:4	0.018 ± 0.001	0.066 ± 0.007(****)	0.025 ± 0.002	0.072 ± 0.006 (****)	0.015 ± 0.002	0.043 ± 0.004 (****)	0.019 ± 0.002	0.046 ± 0.003 (****)

Supplemental Table 3. Data are presented as mean ± standard error of the mean (SEM). The number of samples tested are noted in parentheses. Tukey's Multiple Comparisons Test for Groups. [red] = Statistically lower value than chow diet counterparts of the same sex and genotype; [cyan] = Statistically higher value than chow diet counterparts of the same sex and genotype; [yellow] = Statistically higher value than WT counterparts of the same sex and diet; [green] = Statistically lower than WT counterparts of the same sex and diet. *p value*: $^{*} < 0.05$, $^{**} < 0.01$, $^{***} < 0.001$, $^{****} <$ 0.0001. WT: wild type; KO: knockout; GHS-R: growth; HFD: high-fat diet paradigm.

EFFECTS OF ICV PF-5190457 ON GHRELIN-INDUCED NEURONAL ACTIVATION IN THE ARCUATE NUCLEUS AND OVERNIGHT FOOD INTAKE

We quantified the number of c-Fos immunoreactive (IR) cells in the hypothalamic arcuate nucleus (Arc) of male and female mice treated with ICV ghrelin and PF-5190457. A subset of

mice was anesthetized and perfused 2 hours after treatment, at the end of the 2h food intake experiment. Ghrelin administration increased the number of c-Fos IR cells in both males ($F_{1, 13}$ = 39.13, $p < 0.0001$, **Supplemental Figure S14A**) and females ($F_{1, 13}$ = 48.32, $p < 0.0001$, **Supplemental Figure S14B**). We also detected significant ghrelin x PF-5190457 interactions in males ($F_{1, 13}$ = 7.397, $p < 0.05$, **Supplemental Figure S14A**) and females ($F_{1, 13}$ = 4.79, $p < 0.05$, **Supplemental Figure S14B**). *Post hoc* comparisons indicated that PF-5190457 pretreatment results in lower number c-Fos-IR cells in ghrelin-treated male ($p < 0.01$) and female mice ($p < 0.05$). Interestingly, PF-5190475 did not affect overnight food intake either in male or in female mice, compared to vehicle-treated animals (**Supplemental Figure S14C-D**).

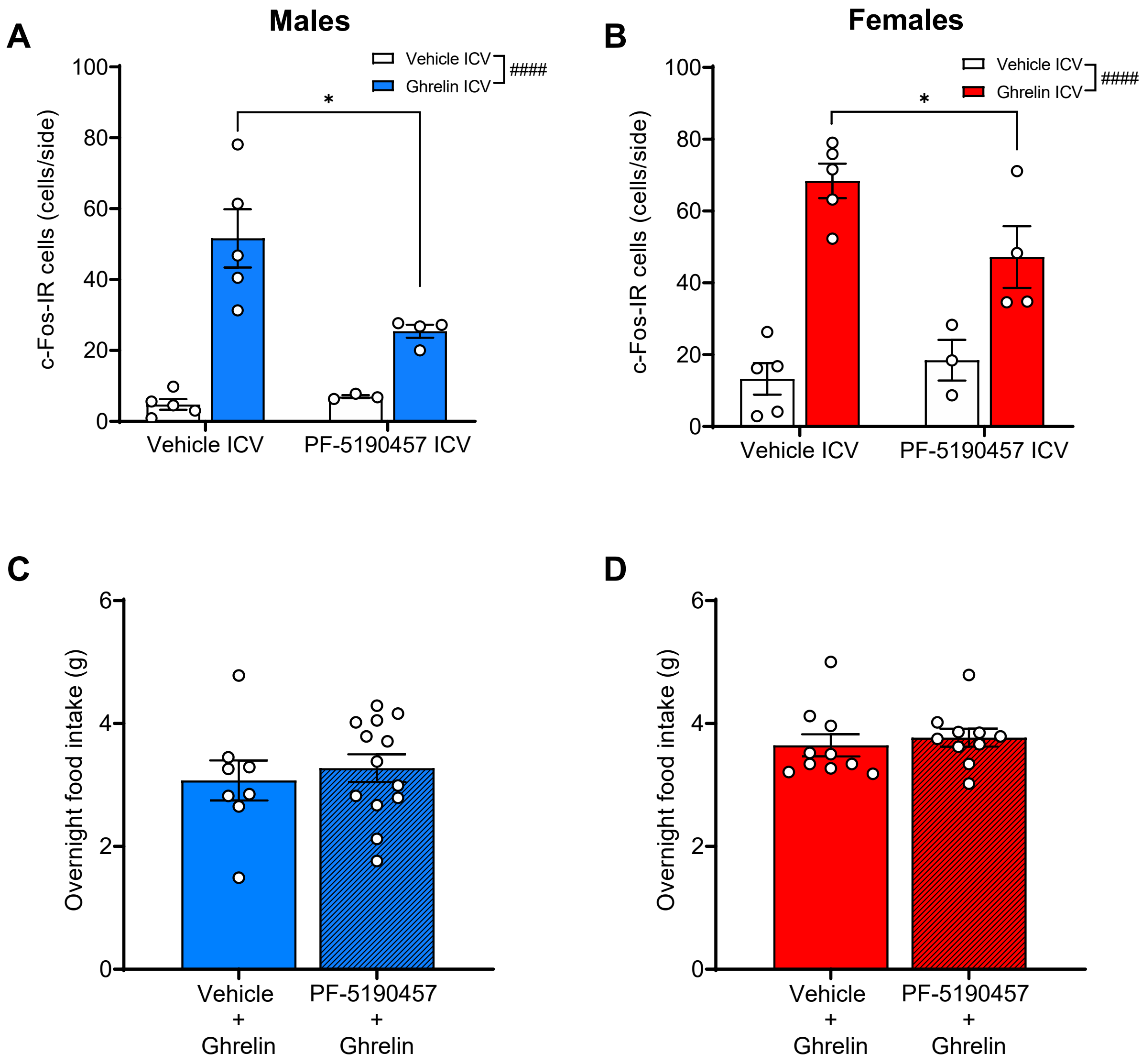

Supplemental Figure S14. PF-5190457 ICV administration attenuated the ghrelin-induced neuronal activation in the arcuate nucleus of the hypothalamus (Arc), but did not have any effect on overnight food intake. (**A**) In male mice, ICV ghrelin significantly increased the number of c-Fos IR cells in the Arc, regardless of vehicle / PF-5190457 pretreatment ($^{\#\#\#\#}p < 0.0001$), but PF-5190457 pretreatment resulted in lower numbers compared to the vehicle ($^{*}p < 0.05$) (vehicle + vehicle: n = 5; PF-5190457 + vehicle: n = 3; ghrelin + vehicle: n = 5; ghrelin + PF-5190457: n = 4). (**B**) In female mice, ICV ghrelin significantly increased the number of c-Fos IR cells in the Arc, regardless of vehicle / PF-5190457 pretreatment ($^{\#\#\#\#}p < 0.0001$), but PF-5190457 pretreatment resulted in lower numbers compared to the vehicle ($^{*}p < 0.05$) (vehicle + vehicle: n = 5; PF-5190457 + vehicle: n = 3; ghrelin + vehicle: n = 5; ghrelin + PF-5190457: n = 4). (**C**) PF-5190457 ICV administration did not affect overnight chow food intake in male mice (vehicle + ghrelin: n = 8; PF-5190457 + ghrelin: n = 13). (**D**) PF-5190457 ICV administration did not affect overnight chow food intake in female mice (vehicle + ghrelin: n = 10; PF-5190457 + ghrelin: n = 10).

ANTI-GHRELIN VACCINE EXPERIMENT IN MALE WISTAR RATS

Generation of Anti-Ghrelin Vaccine Immunization

For the active anti-ghrelin vaccine group, there was a modest escalation of IgG response following the first four doses, unfortunately titers also began to decrease within this immunization timeframe. Specifically, the IgG mid-point titer values obtained for bleeds 1, 2, 3, 4 and 5 were 907, 1185, 1942, 2865, and 1703, respectively **(Supplemental Figure S15B)**. As expected, no antibody response was seen for the sham group (**Supplemental Figure S15A**). The average IgM titer for bleed 1 was 1088, which dropped, as expected, to 530 by bleed 2. Plasma ghrelin affinity (IC_{50}) of the antibody was also monitored, and only a modest affinity (≤ 1331 nM) was observed at bleed 3, which improved slightly by bleed 4 (≤ 311 nM) and 5 (≤ 476 nM; **Supplemental Figure S16**).

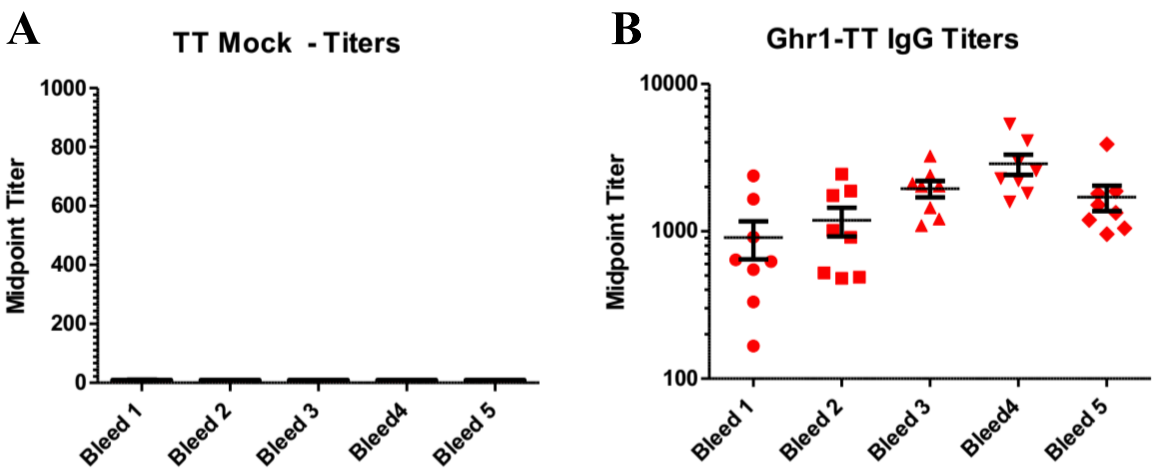

Supplemental Figure S15. Ghrelin (acyl-ghrelin) sequestering antibodies in active vaccine immunized male rats. (**A**) No antibody response was seen for Sham (TT Mock; *n* = 7) male rats. (**B**) Midpoint titers following each immunization boost for Active (Ghr1-TT; *n* = 8) vaccine in male rats.

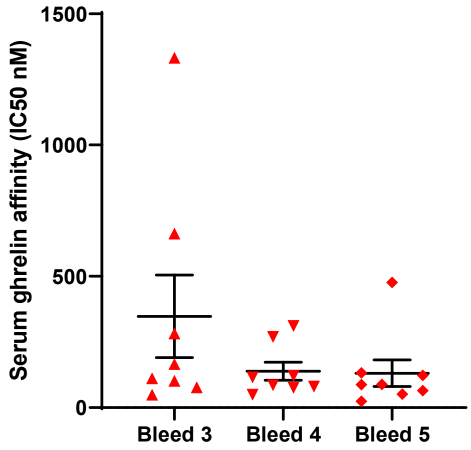

Supplemental Figure S16. Active vaccine immunization resulted in production of modest-affinity anti-ghrelin antibodies. Serum ghrelin affinity is shown as IC_{50} concentration (nM). We could not calculate IC_{50} by bleed 1 and 2, only from bleed 3(Ghr1-TT; n = 8) after active vaccine administration in male rats.

Body Weight

Body weight was recorded from weeks 0-21 starting at the priming dose. A two-way repeated measures ANOVA did not indicate a significant effect of vaccine treatment, but indicated a significant interaction between vaccine treatment and time ($F_{(20, 260)} = 3.535$, $p < 0.0001$) on body weight in male rats. *Post hoc* comparisons did not describe a significant difference between the body weight of vaccine and sham groups at any timepoint. The interaction was significant because of the slower increase in body weight in the sham group (**Supplemental Figure S17**).

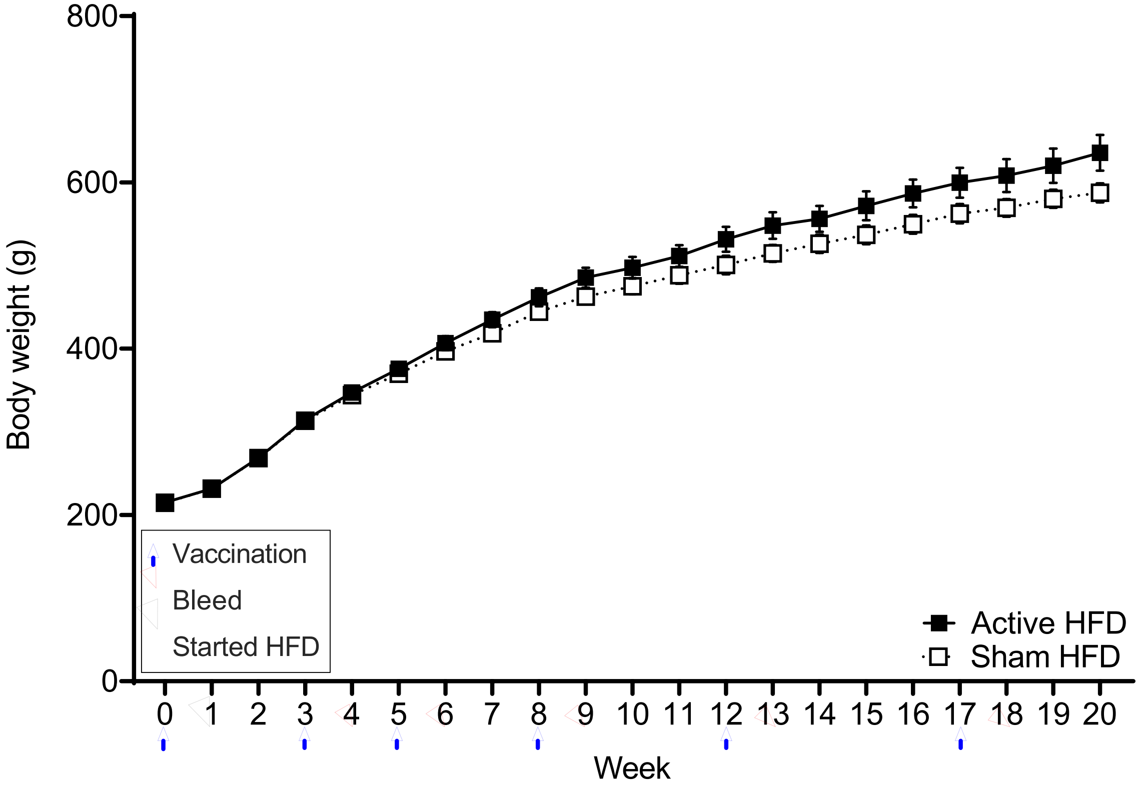

Supplemental Figure S17. Anti-ghrelin vaccine did not blunt body weight gain in actively immunized male rats. Thus, both groups increased their body weight during HFD. Time X Treatment interaction was significant (p < 0.0001), because the increment was lower in the sham treated group during the second half of HFD.

Food Consumption

One week after the vaccine priming dose, all rats started the HFD, which lasted 19 weeks (**Supplemental Figure S18**). A two-way repeated measures ANOVA revealed only a significant effect of time ($F_{(1.617, 21.02)} = 48.22$, $p < 0.0001$), but no effect of vaccine treatment nor Treatment X Time interaction.

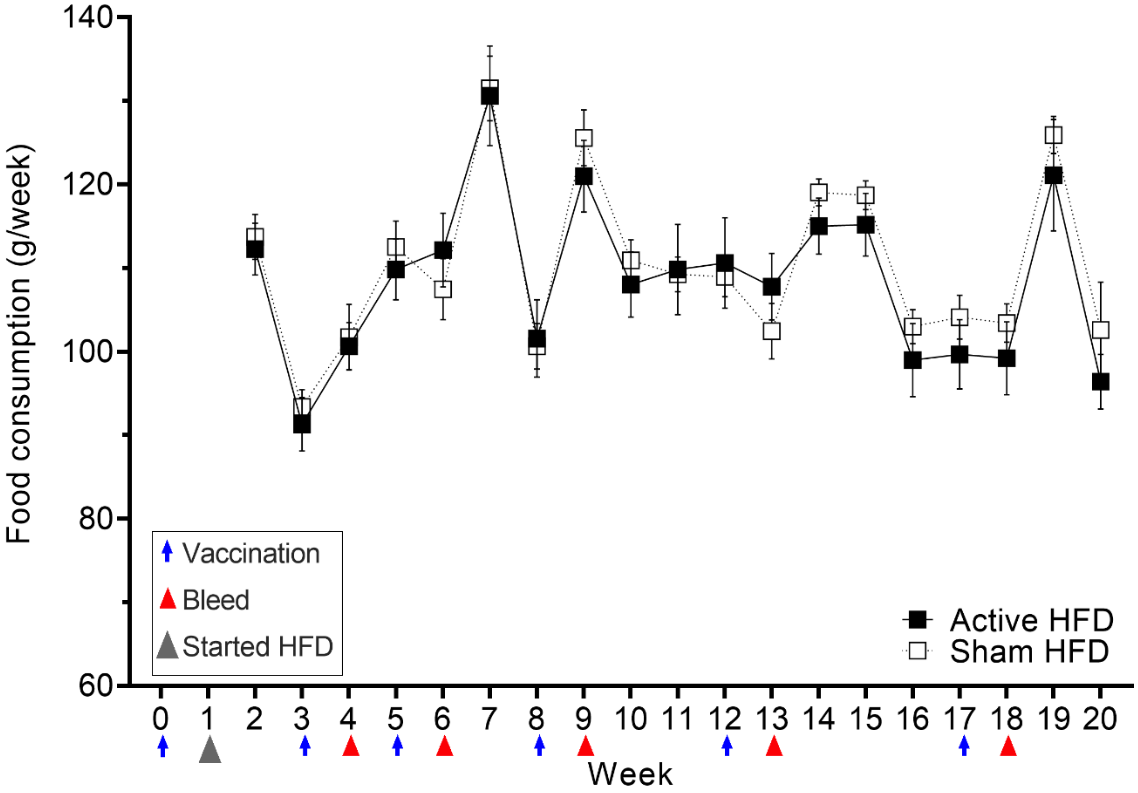

Supplemental Figure S18. Anti-ghrelin vaccine had no effect on weekly food intake. The effect of time was significant, but not the Treatment X Time interaction. Active vaccine immunized group ($n = 8$); sham group ($n = 7$).

Novelty-Suppressed Feeding Test

An unpaired *t*-test revealed no difference in latency to feed (**Supplemental Figure S19A**) between the active vaccine and sham groups. Similarly, no differences were found in caloric intake during the 1 h food consumption upon return to the home cage (**Supplemental Figure S19B**) between the active vaccine and sham groups.

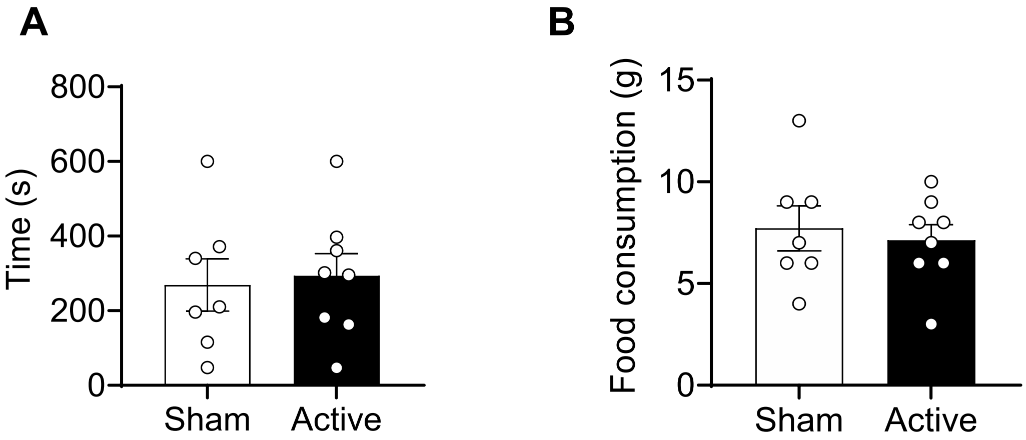

Supplemental Figure S19. Anti-ghrelin vaccine had no effect on suppressed feeding test. (**A**) There was no significant difference in latency to feed during the suppressed feeding test in active vaccine (*n* = 8) and sham (*n* = 7) groups. (**B**) 1 h food consumption in the home cage did not differ between active vaccine and sham groups.

Blood Glucose Concentrations

Student *t*-tests revealed no difference in blood glucose levels during satiety or fasting conditions between the active vaccine and sham groups (**Supplemental Figure S20**).

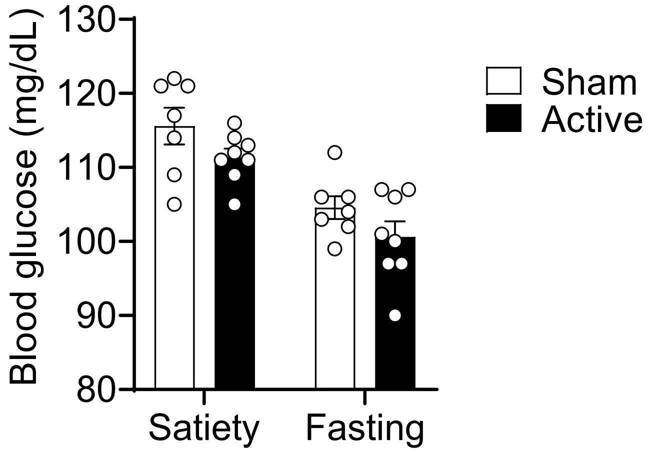

Supplemental Figure S20. Anti-ghrelin vaccine had no effect on blood glucose concentrations on either satiety or fasting conditions. There were no significant differences in blood glucose levels of active vaccine (*n* = 8) and sham (*n* = 7) male rats during either satiety or fasting conditions.

ANTI-GHRELIN VACCINE EXPERIMENT IN MALE AND FEMALE C57BL/6J MICE

Generation of Anti-Ghrelin Vaccine Immunization

As expected, no antibody development was detected for the sham group (**Supplemental Figure S21A**). For the ghrelin-CRM vaccinated mice, the IgG mid-point titer values obtained for bleeds 1, 2, and 3 are 8,695, 12,187, and 10,035, respectively (**Supplemental Figure S21B**).

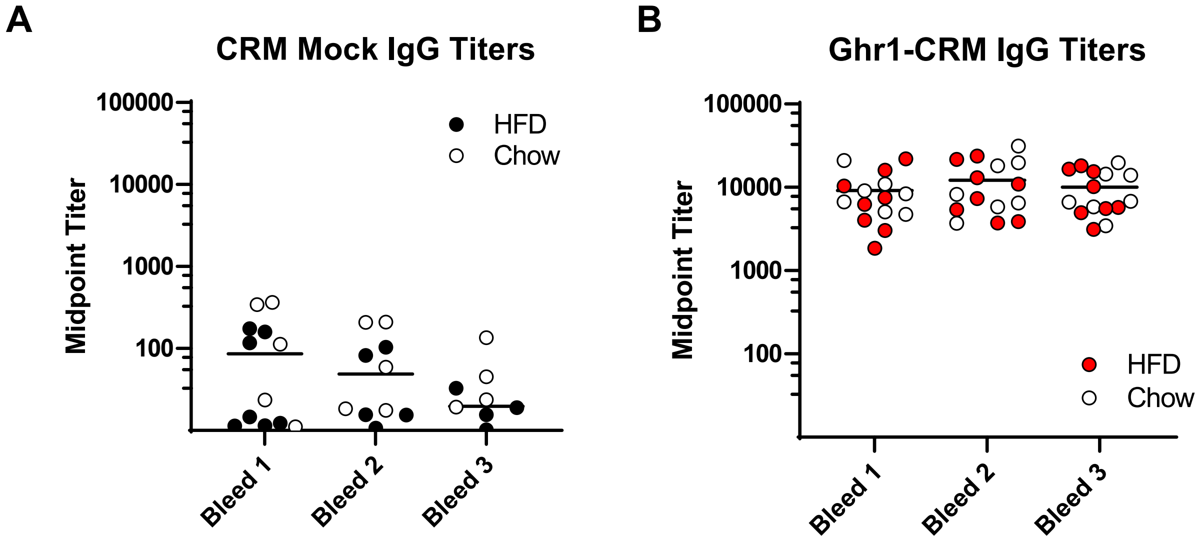

Supplemental Figure S21. Ghrelin (acyl-ghrelin) sequestering antibodies in active vaccine immunized mice. (**A**) No antibody development was seen for Sham (*n* = 16) mice, the average mid-point IgG titers for the mock vaccinated animals were under 200 for all 3 bleeds. (**B**) An immune response was detected with the active vaccine (*n* = 16) mice, as shown by midpoint titers following each immunization boost.

Body Weight

Body weight was recorded from weeks 0-21 starting at the priming dose. The 3-way ANOVA described no difference in body weight gain between the active vaccine and sham groups. Neither the main effect of treatment nor the Treatment x Diet, Treatment x Time, Treatment x Diet x Time interactions were significant. The effect of Time ($F_{1.805, 48.73} = 134.4$, $p < 0.0001$) and Diet ($F_{1, 27} = 19.40$, $p = 0.0002$), and their interaction ($F_{20, 540} = 35.87$, $p < 0.0001$) were significant,

showing that body weight gain was increased in the HFD group compared to a chow diet, regardless of treatment (**Supplemental Figure S22**).

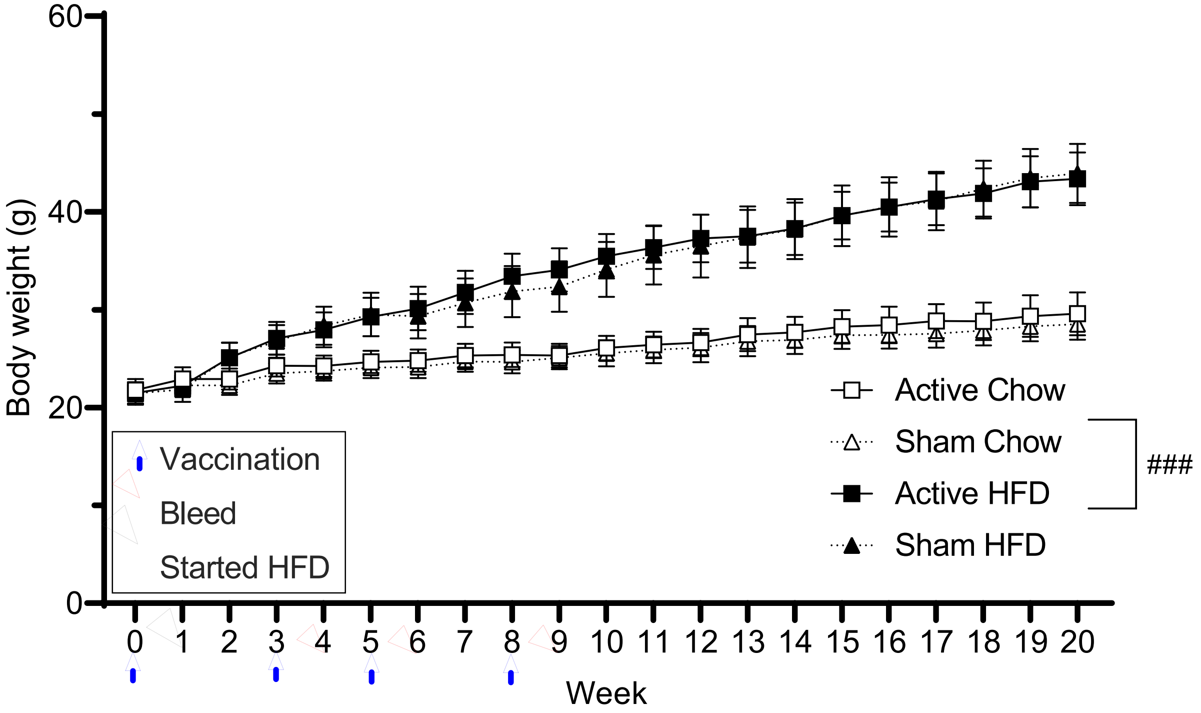

Supplemental Figure S22. Anti-ghrelin vaccine had no effect on body weight gain overtime. The active vaccine (n = 15) and sham groups (n = 16) both significantly gained weight both during HFD and the chow diet. The weight gain with HFD was significantly increased compared to the chow diet ($^{\#\#\#}p = 0.0002$).

Food Consumption

One week after the priming dose, mice were assigned a Chow or HFD paradigm that lasted 19 weeks. Two-way ANOVA did not show any difference in food intake between active vaccine and sham groups, neither with the chow diet nor with HFD (**Supplemental Figure S23**). Only the time effect was significant (chow diet: $F_{2.067, 26.29} = 39.09$, $p < 0.0001$; HFD: $F_{4.531, 58.42} = 6.299$, $p = 0.0002$).

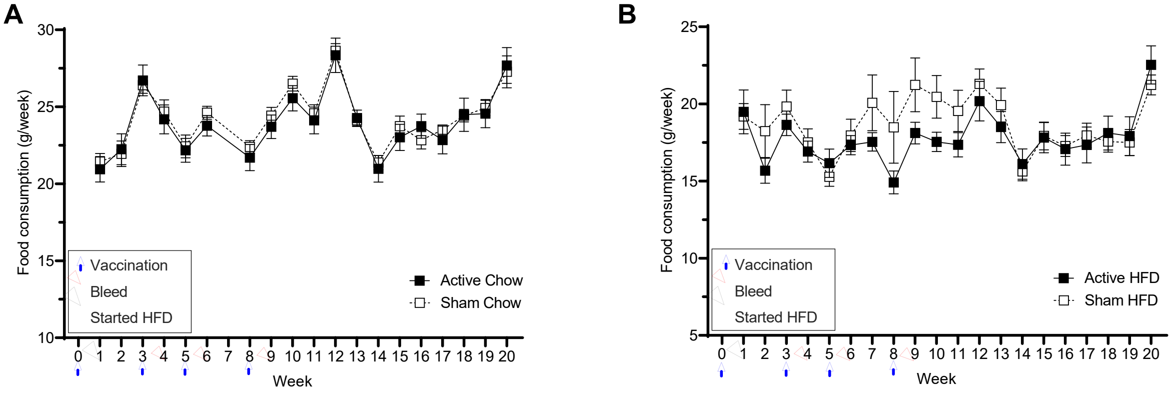

Supplemental Figure S23. Anti-ghrelin vaccine did not affect food consumption overtime. (**A**) Chow diet: There was no difference in food consumption between the active vaccine ($n = 7$) and sham groups ($n = 8$). The effect of time was significant ($p < 0.0001$) (**B**) HFD: There was no difference in food consumption between the active vaccine ($n = 7$) and sham groups ($n = 8$). The effect of time was significant ($p = 0.0002$).

REFERENCES

1 Gould, T. D., Dao, D. T. & Kovacsics, C. E. The open field test. *Mood and anxiety related phenotypes in mice: Characterization using behavioral tests*, 1-20 (2009).

2 Bodnoff, S. R., Suranyi-Cadotte, B., Aitken, D. H., Quirion, R. & Meaney, M. J. The effects of chronic antidepressant treatment in an animal model of anxiety. *Psychopharmacology (Berl)* **95**, 298-302 (1988). https://doi.org:10.1007/bf00181937

3 Thanos, P. K. *et al.* Differences in response to food stimuli in a rat model of obesity: in-vivo assessment of brain glucose metabolism. *Int J Obes (Lond)* **32**, 1171-1179 (2008). https://doi.org:10.1038/ijo.2008.50

4 Cai, N. S. *et al.* Opioid-galanin receptor heteromers mediate the dopaminergic effects of opioids. *J Clin Invest* **129**, 2730-2744 (2019). https://doi.org:10.1172/jci126912

5 Fischer, A. H., Jacobson, K. A., Rose, J. & Zeller, R. Hematoxylin and eosin staining of tissue and cell sections. *CSH Protoc* **2008**, pdb.prot4986 (2008). https://doi.org:10.1101/pdb.prot4986

6 Subramanian, A. *et al.* Gene set enrichment analysis: a knowledge-based approach for interpreting genome-wide expression profiles. *Proc Natl Acad Sci U S A* **102**, 15545-15550 (2005). https://doi.org:10.1073/pnas.0506580102

7 Li, B. & Dewey, C. N. RSEM: accurate transcript quantification from RNA-Seq data with or without a reference genome. *BMC Bioinformatics* **12**, 323 (2011). https://doi.org:10.1186/1471-2105-12-323

8 Langmead, B. & Salzberg, S. L. Fast gapped-read alignment with Bowtie 2. *Nat Methods* **9**, 357-359 (2012). https://doi.org:10.1038/nmeth.1923

9 Love, M. I., Huber, W. & Anders, S. Moderated estimation of fold change and dispersion for RNA-seq data with DESeq2. *Genome Biol* **15**, 550 (2014). https://doi.org:10.1186/s13059-014-0550-8

10 Zigman, J. M. *et al.* Mice lacking ghrelin receptors resist the development of diet-induced obesity. *J Clin Invest* **115**, 3564-3572 (2005). https://doi.org:10.1172/jci26002

11 Cornejo, M. P. *et al.* Ghrelin treatment induces rapid and delayed increments of food intake: a heuristic model to explain ghrelin's orexigenic effects. *Cellular and Molecular Life Sciences* **78**, 6689-6708 (2021). https://doi.org:10.1007/s00018-021-03937-0

12 Wenthur, C. J. *et al.* Ghrelin Receptor Influence on Cocaine Reward is Not Directly Dependent on Peripheral Acyl-Ghrelin. *Sci Rep* **9**, 1841 (2019). https://doi.org:10.1038/s41598-019-38549-z

13 Zallar, L. J. *et al.* Development and initial characterization of a novel ghrelin receptor CRISPR/Cas9 knockout wistar rat model. *Int J Obes (Lond)* **43**, 344-354 (2019). https://doi.org:10.1038/s41366-018-0013-5

14 Börchers, S. *et al.* From an Empty Stomach to Anxiolysis: Molecular and Behavioral Assessment of Sex Differences in the Ghrelin Axis of Rats. *Front Endocrinol (Lausanne)* **13**, 901669 (2022). https://doi.org:10.3389/fendo.2022.901669
