# Supplementary material for: Genetic or pharmacological GHSR blockade has sexually dimorphic effects in rodents on a high-fat diet
Source: Commun Biol. 2024 May 25;7:632. doi: 10.1038/s42003-024-06303-5 (PMC11127961; doi:10.1038/s42003-024-06303-5)
Supplement: Supplementary file 3 — Description of Additional Supplementary Files [file 42003_2024_6303_MOESM3_ESM.pdf]

Description of Additional Supplementary Files

File name: Supplementary Data 1

Description: The source data behind the graphs in the paper

File name: Supplementary Data 2

Description: GSEA results for homozygous GHSR-KO with high fat diet vs. wildtype with high fat diet. Genes were ranked by log2 fold change values, and GSEA was run with 10,000 gene set permutations. Significance values listed as zero indicate less than 1/10,000.

File name: Supplementary Data 3

Description: GSEA results for homozygous GHSR-KO with normal chow diet vs. wildtype with normal chow diet. Genes were ranked by log2 fold change values, and GSEA was run with 10,000 gene set permutations. Significance values listed as zero indicate less than 1/10,000.

File name: Supplementary Data 4

Description: For comparing the two genotypes with the expression normalized to the control diet, mean differences of the (normalized counts)/(average of normalized counts over the corresponding control chow diet) were ranked by the GSEA software for genes with average expression greater than 100 DESeq2 normalized counts. Significance values listed as zero indicate less than 1/10,000.

File name: Supplementary Data 5

Description: GSEA results for wildtype with high fat diet vs. wildtype with normal chow diet. Genes were ranked by log2 fold change values, and GSEA was run with 10,000 gene set permutations. Significance values listed as zero indicate less than 1/10,000.

File name: Supplementary Data 6

Description: GSEA results for GHSR-KO with high fat diet vs. GHSR-KO with normal chow diet. Genes were ranked by log2 fold change values, and GSEA was run with 10,000 gene set permutations. Significance values listed as zero indicate less than 1/10,000.

File name: Supplementary Data 7

Description: GSEA results for homozygous GHSR-KO with high fat diet vs. wildtype with high fat diet. Genes were ranked by log2 fold change values, and GSEA was run with 10,000 gene set permutations. Significance values listed as zero indicate less than 1/10,000.

File name: Supplementary Data 8

Description: GSEA results for homozygous GHSR-KO with normal chow diet vs.wildtype with normal chow diet. Genes were ranked by log2 fold change values, and GSEA was run with 10,000 gene set permutations. Significance values listed as zero indicate less than 1/10,000.

File name: Supplementary Data 9

Description: GSEA results for wildtype with high fat diet vs. wildtype with normal chow diet. Genes were ranked by log2 fold change values, and GSEA was run with 10,000 gene set permutations. Significance values listed as zero indicate less than 1/10,000.

File name: Supplementary Data 10

Description: GSEA results for GHSR-KO with high fat diet vs. GHSR-KO with normal chow diet. Genes were ranked by log2 fold change values, and GSEA was run with 10,000 gene set permutations. Significance values listed as zero indicate less than 1/10,000.
